# Occlusal outcome of orthodontic treatment: a systematic review with meta-analyses of randomized trials

**DOI:** 10.1093/ejo/cjae060

**Published:** 2024-11-28

**Authors:** Spyridon N Papageorgiou, Theodora Giannakopoulou, Theodore Eliades, Vaska Vandevska-Radunovic

**Affiliations:** Clinic of Orthodontics and Pediatric Dentistry, Center for Dental Medicine, University of Zurich, Plattenstrasse 11, 8032 Zurich, Switzerland; Department of Paediatric Oral Heath and Orthodontics, University Centre for Dental Medicine UZB, University of Basel, Mattenstrasse 40, 4058 Basel, Switzerland; Clinic of Orthodontics and Pediatric Dentistry, Center for Dental Medicine, University of Zurich, Plattenstrasse 11, 8032 Zurich, Switzerland; Department of Orthodontics, Institute of Clinical Dentistry, Faculty of Dentistry, University of Oslo, P.O. Box 1072 Blindern, N-0316 Oslo, Norway

**Keywords:** orthodontic treatment, fixed appliance, treatment effectiveness, randomized clinical trial, systematic review, evidence-based medicine

## Abstract

**Background:**

Several appliances or treatment protocols are marketed to either patients or orthodontists as being associated with improved orthodontic outcomes. However, clinical decision-making should be based on robust scientific evidence and not marketing claims or anecdotal evidence.

**Objective:**

To identify appliances/protocols being associated with improved outcomes of fixed appliance treatment.

**Search methods:**

Unrestricted literature searches in seven databases/registers for human studies until March 2024.

**Selection criteria:**

Randomized or quasi-randomized clinical trials on human patients of any age, sex, or ethnicity receiving comprehensive orthodontic treatment with fixed appliances and assessing occlusal outcome with either the Peer Assessment Rating (PAR) or the American Board of Orthodontics-Objective Grading System (ABO-OGS) index.

**Data collection and analysis:**

Duplicate/independent study selection, data extraction, and risk of bias assessment with the Cochrane RoB 2 tool. Random-effects meta-analyses of averages or mean differences with their 95% Confidence Intervals (CI), followed by meta-regression/subgroup/sensitivity analyses and assessment of the quality of clinical recommendations with the Grades of Recommendations, Assessment, Development, and Evaluation (GRADE) approach.

**Results:**

Data from 20 small- to moderately-sized trials covering 1470 patients indicated that orthodontic treatment with fixed appliances is effective and results on average in a final PAR score of 6.0 points (95% CI 3.9–8.2 points), an absolute PAR reduction of 23.0 points (95% CI 15.6–30.4 points), a % PAR reduction of 82.6% (95% CI 70.8%–94.4%), and an absolute ABO-OGS score of 18.9 points (95% CI 11.7–26.2 points). However, very high between-study heterogeneity (*I*^2^ > 75%) was seen for both PAR and ABO-OGS. Extraction treatment was associated with significantly better occlusal outcome than non-extraction treatment with ABO-OGS (12.9 versus 16.6 points; *P* = .02). There was no statistically significant difference in occlusal outcome with (i) 0.018″-slot or 0.022″-slot brackets; (ii) customized or prefabricated brackets; (iii) anchorage reinforcement with temporary anchorage devices; (iv) use of vibrational adjuncts; and (v) aligners or fixed appliances (*P* > .05 in all instances), while small benefits were seen with indirectly bonded brackets.

**Conclusions:**

Considerable between-study heterogeneity exists in the reported occlusal outcome of fixed appliance treatment, and different appliances or adjuncts have little effect on this. Standardization and/or automatization of the scoring procedures for PAR and ABO-OGS might help to improve consistency and reliability of outcome measurement in orthodontic trials.

**Registration:**

PROSPERO (CRD42024525088).

## Introduction

Orthodontic treatment with fixed appliances is the gold standard for correcting malocclusions of varying severity, as they provide adequate control of tooth movement in all three dimensions. At the same time, there exists a wide range of different fixed appliances, adjuncts, and clinical protocols that have been proposed to perform better than others [1–4]. However, such claims from researchers or commercial companies need to be substantiated by high-quality clinical evidence and not by marketing claims or uncontrolled evidence [5, 6].

The outcome of orthodontic treatment can be objectively assessed through the patient’s records according to a set of predefined criteria. The Peer Assessment Rating (PAR) is one of the most commonly used indices to assess treatment outcome in an objective and reproducible way [7], which scores various occlusal traits that make up a malocclusion. The PAR index contains various items (contact point displacement, buccal occlusion, overjet, overbite, and centerline), which are scored, weighted, and added up to make up a total sum. Scoring of dental cast models with the PAR index requires the use of a specific ruler, requires calibration of the examiner, can be measured before and/or after treatment, and can be expressed as absolute post-treatment value, absolute treatment-related reduction, or % treatment-related reduction. Another index to measure the occlusal outcome of orthodontic treatment is the American Board of Orthodontic (ABO) Objective Grading System (OGS) [8]. The ABO-OGS index scores eight criteria that contribute to ideal intercuspation and function: alignment/rotations, marginal ridges, buccolingual inclination, overjet, occlusal contacts, occlusal relationships, interproximal contacts, and root angulation. The first eight criteria are assessed from the dental models, while the last one is assessed from orthopantomograms. Ideal occlusion and alignment receive a score of 0 points, while deviations from this are given penalty points. Consequently, a high percentage of accordance can be achieved in both interexaminer and intraexaminer assessments after proper calibration [9]. In addition to functioning as an objective clinical examination tool for ABO, the ABO-OGS can also be used for the assessment of treatment progress and final outcomes with increased reliability, validity, and precision [10]. Contrary to PAR, the ABO-OGS is not supposed to be used to assess baseline malocclusion severity, and instead the ABO Discrepancy Index (DI) has been developed for this purpose [11].

Even though both PAR and ABO-OGS are used widely both for clinical and research purposes, they are two distinct indices that correlate poorly—regardless of if absolute PAR values or PAR reductions are used [12–14]. However, both are used extensively in research to assess both the outcome of fixed appliance treatment and post-debond longitudinal occlusal changes [15–18]. Additionally, it is important to have a benchmark about occlusal outcomes in a future experimental clinical setting to be used when designing future trials and performing sample size calculations, as well as develop a core outcome set relevant to both orthodontists and patients in order to minimize the use of surrogate endpoints of little clinical relevance [19].

### Objective

The aim of this systematic review was to identify and critically appraise evidence from randomized trials using either PAR or ABO-OGS to assess the occlusal outcome of comprehensive fixed appliance treatment to provide a benchmark for future studies. The secondary aim of this review was to assess the effect of different appliances, protocols, or adjuncts on the occlusal outcome of comprehensive fixed appliances treatment.

## Materials and methods

### Protocol and registration

The protocol for this review was developed a priori, registered in PROSPERO (CRD42024525088), and all deviations were reported (Supplementary Appendix 1). This review was conducted according to the Cochrane Handbook for Systematic Reviews of Interventions [20] and is reported according to the Preferred Reporting Items for Systematic Reviews and Meta-Analyses (PRISMA) statement [21].

### Eligibility criteria

Eligible to be included in this review were clinical trials on systemically healthy patients of any age/sex/ethnicity, with any kind of malocclusion treated with comprehensive orthodontic treatment with fixed appliances of any type placed on all teeth (at least up to the first permanent molar) of both jaws. Included were randomized or quasi-randomized trials of parallel design on humans, while excluded were non-randomized studies, animal studies, and non-clinical studies. Excluded were studies on patients with systemic diseases, patients receiving first-phase orthopedic treatment or orthognathic treatment, patients receiving partial treatment or treatment only with removable appliances, and studies not using PAR or ABO-OGS. The review’s primary outcome was the occlusal outcome of orthodontic treatment, assessed as either absolute PAR values or total ABO-OGS values post-treatment. Secondary outcomes included: (i) absolute treatment-related PAR reduction (pre-treatment minus post-treatment); (ii) % treatment-related PAR reduction; (iii) post-treatment values for each separate ABO-OGS component (alignment, marginal ridges, buccolingual inclination, overjet, occlusal contacts, occlusal relationships, interproximal contacts, and root angulation); and (iv) treatment duration in months. Included were all clinical settings to increase the generalizability of this review’s results.

### Information sources and search

Seven databases were searched without restrictions for publication language/year/type from inception up to March 2024 (Supplementary Appendix 2). The reference lists and citation lists through Google Scholar of eligible articles and existing systematic reviews were manually reviewed to identify any potentially relevant studies to include.

### Selection process

Initially, the title or abstract of identified studies was checked to eliminate obviously irrelevant to the review studies. Subsequently, the full text of all remaining studies was checked against the review’s eligibility criteria for potential inclusion. Study selection was performed in duplicate (SNP/TG) and independently, while any disagreements were resolved through discussion with a third author (TE).

### Data collection process and items

Data collection utilized a pre-defined and piloted extraction form, encompassing the following data: (i) study characteristics, including the first author with the year of publication, study design, and clinical setting (country); (ii) patient characteristics, comprising age and sex; (iii) sample size; (iv) trial scope and experimental groups; (v) measurements of baseline malocclusion severity; (vi) percentage of tooth extractions; and (vii) outcomes assessed. Data extraction was likewise performed independently by two authors (SNP/TG), while any disparities were resolved through discussion with a third author (ME). In case of missing data, the corresponding authors were contacted to request this data or individual patient data.

### Risk of bias of individual studies

The risk of bias of all included trials was assessed with the Cochrane risk of bias in randomized trials (RoB 2) tool [22] based on post-treatment occlusal outcome measurement. All assessments were performed by two authors independently (SNP/TG), with discrepancies resolved through discussion with a third author (TE).

### Effect measures and data synthesis

A single-group meta-analysis for pooled average outcomes (one-group pooling) was undertaken, followed by a pairwise meta-analysis (two-group comparison) with Mean Difference (MD) and their corresponding 95% Confidence Intervals (CI). As treatment outcome was expected to vary considerably (according to different patient demographics, baseline severity, and treatment-related characteristics [23–26]), a random-effects model was chosen a priori as more appropriate to express this variability and calculate the average distribution of treatment effects across studies [27]. A restricted maximum likelihood variance estimator was chosen [28], while the Knapp & Hartung adjustment [29] was used for meta-analyses with > 3 studies. Between-studies heterogeneity was investigated by visually inspecting forest plots or by estimating *τ*^2^ (absolute measured heterogeneity) and *I*^2^ (realative estimated inconsistency) with their 95% uncertainty intervals. Random-effects predictions were estimated for meta-analyses with ≥ 3 studies to incorporate observed heterogeneity and interpretate the meta-analytical estimates by providing a range of expected effects across various future clinical settings [30]. Random-effects meta-regression / subgroup analyses were used to investigate the effect of mean age, patient sex (through the % of male patients in the sample), baseline severity, % of tooth extractions, and treatment duration on treatment outcomes (Supplementary Appendix 1). All analyses were conducted in R 4.2.2. (R Foundation for Statistical Computing, Vienna, Austria) by one author (SNP), with open dataset [31], alpha set at 0.05 and a two-sided *P*-value.

### Reporting bias assessment and certainty assessment

Reporting biases (including small-study effects and the possibility of publication bias) were assessed through plotting contour-enhanced funnel plots and testing with Thompson’s method for meta-analyses with at least 10 studies. The Grades of Recommendations, Assessment, Development, and Evaluation (GRADE) approach was used to assess the quality of clinical recommendations from pairwise (two-group) meta-analyses [32]. The GRADE approach was not used for the one-group pooled prevalences since no formal guidance exists.

## Results

### Study selection

**Figure 1.**
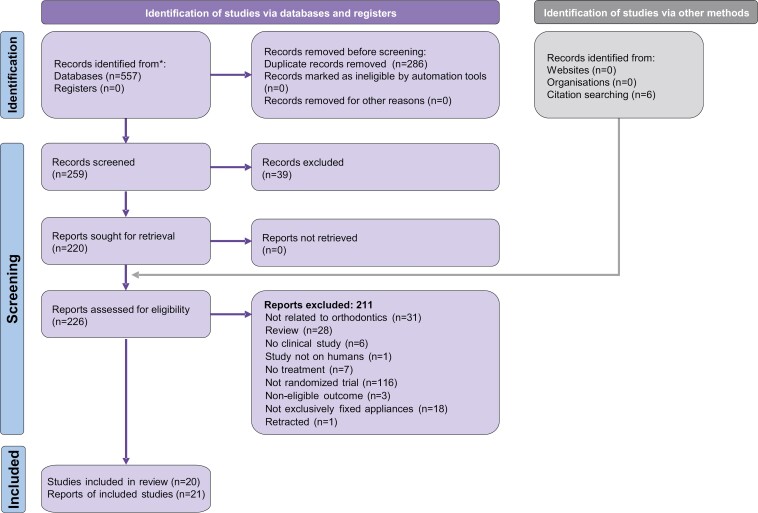
PRISMA flow diagram for the identification and selection of eligible studies.

The initial electronic database search yielded 557 records, and six additional were identified through manual searching (Fig. 1). After eliminating 286 duplicates, 259 records were left for further evaluation and were assessed against the eligibility criteria (Supplementary Appendix 3). Ultimately, 21 publications, corresponding to 20 distinct trials, were included in the quantitative synthesis (with one trial being reported in two publications [33, 34]). Most (19/20) were published as journal papers, while one was published as a master’s thesis with no subsequent journal publication [35]. All were in English, except for one published in Chinese [36]. The corresponding authors of eight included studies were contacted to request additional data or datasets, but only three responded (Supplementary Appendix 4) and provided data.

### Study characteristics

The characteristics of included studies are summarized in Table 1. Among the 20 included studies, the majority (85%; 17/20) were randomized, while the remaining three employed either partially random allocation methods or included groups from previous randomized trials. These studies were conducted in university clinics, hospitals, or private practices from twelve different countries (Belgium, China, Germany, Great Britain, Greece, India, Iran, South Korea, Syria, the Netherlands, the United States of America, and Turkey). In total, 1470 patients were included in the 20 studies (median 54 patients/trial), who were 38% male (413/1074; from the 15 studies reporting sex) and were on average 18.4 years of age (from the 18 studies reporting on age). Among the included trials, the majority (55%; 11/20) compared different types of fixed appliances, two assessed different anchorage reinforcement methods [37, 38], and one assessed vibrational adjunct to fixed appliance treatment [39]. Three trials compared fixed appliances to aligners [35, 40, 41], from which only the former group was used for the one-group meta-analyses of average outcomes. Three trials other trials assessed different retention methods after removal of the fixed appliances [17, 42, 43], but reported the occlusal outcome at debond, which was used in the analyses. Baseline malocclusion severity, either with PAR or ABO-DI, was assessed in 11 trials, while another four reported baseline irregularity levels (that were however disregarded here). As far as extractions are concerned, 40% of the trials (*n* = 8) included only non-extraction cases, 20% (*n* = 3) only extraction cases, 25% (*n* = 4) extraction/non-extraction cases, and 15% (*n* = 3) did not report whether extractions were performed or not. The PAR index after treatment (either are absolute posttreatment values, absolute treatment-related reduction, or % absolute treatment-related reduction) was used in 11 trials with the British weighting. The ABO-OGS index was used post-treatment in 10 trials, with eight trials assessing all (8/8) of its components, one trial [34] excluding root parallelism, and one trial [35] reporting only 2/8 of the ABO-OGS components (marginal ridges and buccolingual inclination). Some studies also reported ABO-OGS scores pre-treatment, but as this is not described in the instrument’s guidance (which uses instead the ABO-DI), this was omitted here. Finally, treatment duration was reported in 10 of the included trials.

**Table 1.** Characteristics of included studies.

Study	Design; source; country^a^	Patients (M/F); age	Scope	Groups	Malocclusion	Ex	Severity index: baseline value	Outcomes
Abo Ayach 2021	RCT; Uni; SY	54 (25/29); 20.5	Retention	G1: BLR G2: VFR G3: VFR + LLLT	Moderate crowding; skeletal Class I; “normal” growth pattern	0%	-	ABO-OGS
DiBiase 2011	RCT; Hosp/Uni; GB	62 (32/30); 16.3	FXA	G1: Labial FXA G2: Labial SL FXA	LII 5-12; no complete overbite	100%	PAR_GB-weight_: 39.4	PAR_GB-weight_; TxDur
DiBiase 2018	RCT; Hosp/Uni; GB	61 (30/31); 13.9	FXA	G1: Labial FXA G2: Labial FXA + VIB G3: Labial FXA + PLB	Mandibular crowding	100%	PAR_GB-weight_: 33.9 LII: 7.8	PAR_GB-weight_; TxDur
Fleming 2010	RCT; Hosp/Uni; GB	54 (18/36); 15.8	FXA	G1: Labial FXA G2: Labial SL FXA	Mild mandibular crowding	27.8%	PAR_GB-weight_: 30.9	PAR_GB-weight_; TxDur
Hegele 2021	qRCT; Uni; DE	38 (15/23); 14.3	FXA	G1: Labial SL FXA G2: Labial CAD SL FXA	No orthognathic cases; Class I	0%	LII: 3.6	ABO-OGS
Jaber 2023	RCT; Uni; SYR	40 (14/26); 21.4	FXA/aligners	G1: Labial FXA G2: Aligners	Class I molars; LII > 6.0; ABO-DI ≥ 25.0; no hypodontia	100%	ABO-DI: 32.6	ABO-OGS
Jackers 2021	qRCT^b^; Uni; BE	24 (7/17); 23.4	FXA	G1: Labial SL FXA G2: Labial CAD SL FXA	LII ≤ 6.0	NR	ABO-DI: 14.1 LII: 2.9	ABO-OGS
Jung 2021	qRCT^c^; Pract; KR	134 (37/97); 22.7	FXA	G1: Labial Cer FXA G2: Labial SL Cer FXA	-	71.6%	ABO-DI: 14.3 LII: 4.4	ABO-OGS; TxDur
Kaklamanos 2917	RCT; Uni; GR	22 (NR); 14.2	FXA	G1: Labial FXA G2: Labial SL FXA	Class I; LII ≤ 9.0	0%	PAR_NR_: 17.6	PAR_NR_; TxDur
Kumar 2011	RCT; Uni; IN	224 (NR); NR	Retention	G1: Essig retainer G2: Begg retainer	Posttreatment	NR	-	PAR_NR_
Lin 2022	RCT; Uni; US	54 (NR); 26.3	FXA/aligners	G1: Labial FXA G2: Aligners	Class I canine/molars; LII ≤ 4.0; no crossbite/openbite/impaction	0%	-	ABO-OGS; TxDur
Liu 2022	RCT; Uni; CN	71 (32/39); 21.5	FXA	G1: Labial SL FXA G2: Labial CAD SL FXA	Class I-III	0%	PAR_NR_: 20.6	PAR_NR_; TxDur
Moslemzadeh 2018	RCT; Pract; IR	66 (NR); 19.3	Retention	G1: VFR (1.0 mm) G2: VFR (1.5 mm) G3: Hawley retainer	Posttreatment; optimal occlusion	NR	-	ABO-OGS
Penning 2017	RCT; Uni; NL	180 (77/103); 14.1	FXA	G1: Labial SL FXA G2: Labial CAD SL FXA	Class I–III (≤half-cusp)	0%	PAR_GB-weight_: 22.6	PAR_GB-weight_; TxDur
Preston 2017	RCT; Uni; US	44 (17/27); 26.6	FXA/aligners	G1: Labial FXA G2: Aligners	Mild crowding; Class I; no hypodontia	0%	-	ABO-OGS (partial)
Sandler 2008	RCT; Hosp; GB	51 (13/38); 15.2	FXA	G1: Labial FXA + HG G2: Labial FXA + TAD	Class I–II; maximum anchorage need	5.9%	PAR_GB-weight_: 35.9	PAR_GB-weight_; TxDur
Sandler 2014	RCT; Hosp; GB	78 (41/37); 14.2	FXA	G1: Labial FXA + HG G2: Labial FXA + Nance G3: Labial FXA + TAD	Maximum anchorage need; no hypodontia	98.7%	PAR_GB-weight_: 35.1	PAR_GB-weight_; TxDur
Sharma 2010	RCT; Uni; IN	30 (NR); NR	FXA	G1: Labial FXA (Begg) G2: Labial FXA (modified Begg) G3: Labial FXA (straightwire)	Crowding; incisor proclination; anchorage reinforcement need	100%	-	PAR_GB-weight_
Yassir 2019_coll_	RCT; Hosp; GB	153 (48/105); 19.0	FXA	G1: Labial FXA (0.022” slot) G2: Labial FXA (0.018” slot)	Moderate-complex cases (via IOTN); no hypodontia; no orthognathic cases	73.2%	-	ABO-OGS; PAR_GB-weight_
Yildirim 2018	RCT; Uni; TR	30 (7/23); 15.7	FXA	G1: Labial FXA (directly bonded) G2: Labial FXA (indirectly bonded)	Mild/moderate crowding; Class I canines/molars; no skeletal discrepancy	0%	-	ABO-OGS

ABO, American Board of Orthodontics; BLR, bonded lingual/palatal retainer; CAD, computer-assisted designed/manufactured; Cer, ceramic; coll, multiple trial reports collated; DI, discrepancy index; Ex, extraction treatment; FXA, conventional fixed appliances (brackets); Hosp, hospital; IOTN, index of orthodontic treatment need; LII, little’s irregularity index (mandibular); LLLT, low-level laser therapy; M/F, male/female; Non-Ex, non-extraction; NR, not reported; OGS, objective grading system; PAR, peer assessment rating; PLB, placebo; PM-Ex, premolar extraction; Pract, private practice; qRCT; quasi-randomized clinical trial; RCT, randomized clinical trial (of parallel design); SL, self-ligating; TAD, temporary anchorage device; Tx, treatment; TxDur, treatment duration; Uni, university/college clinic; VFR, vacuum formed retainer; VIB, vibration supplement.

^a^country given its ISO-2 name.

^b^one arm originating from a randomized trial and another from a retrospective analysis of records.

^c^patients were randomized only if they didn’t have a treatment preference.

### Risk of bias of included studies

The risk of bias of included trials with the RoB 2 tool is presented in Supplementary Appendix 5. Issues with the randomization process were found for 8/20 trials: two used quasi-randomization, one included a previously randomized trial arm, three randomized patients to retention and not treatment, while two trials showed baseline imbalances, which could be due to ineffective randomization. Issues with the measurement of the occlusal outcome were found for 12/20 trials, including either lack of blinding (10/20 trials) or lack of assessor’s training/calibration (12/20). Finally, even though some trials reported being registered, no trial reported having an a priori statistical analysis plan that could be used to compare planned versus performed analyses.

### Data synthesis

Synthesis of available evidence from included trials (Supplementary Appendix 6) is reported as single-group meta-analyses of pooled average treatment outcomes (Table 2), pairwise (two-group) meta-analyses of MDs (Table 3), or data reported from single trials (Supplementary Appendix 7).

**Table 2.** Single-group meta-analyses of averages.

Outcome	*n*	Mean (95% CI)	*τ* ^2^ (95% CI)	*I* ^2^ (95% CI)	Prediction
PAR postTx	10	6.03 (3.88, 8.18)	8.68 (3.96, 29.99)	100% (99%, 100%)	0, 13.17
PAR reduction	5	22.97 (15.57, 30.37)	32.92 (10.22, > 100.00)	96% (93%, 98%)	2.83, 43.12
% PAR reduction	6	82.59 (70.80, 94.39)	>100.00 (47.00, >100.00)	99% (99%, 100%)	49.13, 100.00
ABO-OGS total post-Tx	7	18.90 (11.65, 26.15)	60.61 (24.51, >100.00)	99% (99%, 99%)	0, 40.32
ABO-OGS: alignment/rotations	6	3.26 (0.20, 6.31)	8.43 (3.24, 50.78)	99% (99%, 100%)	0, 11.98
ABO-OGS: marginal ridges	6	2.74 (1.08, 4.39)	2.42 (0.89, 14.69)	99% (98%, 99%)	0, 7.41
ABO-OGS: buccolingual inclination	6	3.21 (1.48, 4.93)	2.63 (0.98, 16.09)	98% (97%, 99%)	0, 8.09
ABO-OGS: overjet	6	1.96 (0.39, 3.53)	2.15 (0.75, 12.90)	97% (95%, 98%)	0, 6.38
ABO-OGS: occlusal contacts	6	3.72 (1.14, 6.30)	5.84 (2.16, 36.17)	98% (97%, 99%)	0, 10.99
ABO-OGS: occlusal relationships	6	2.18 (0.45, 3.92)	2.67 (0.97, 15.74)	99% (98%, 99%)	0, 7.11
ABO-OGS: interproximal contacts	6	0.19 (0, 0.51)	0.06 (0.02, 0.88)	85% (68%, 93%)	0, 0.94
ABO-OGS: root angulation	5	1.29 (0.48, 2.11)	0.37 (0.09, 3.55)	89% (77%, 95%)	0, 3.44
Tx duration	14	21.96 (17.54, 26.38)	57.82 (29.60, >100.00)	100% (100%, 100%)	4.79, 39.12

ABO, American Board of Orthodontics; OGS, objective grading system; PAR, peer assessment rating; Tx, treatment.

**Table 3.** Pairwise (two-group) meta-analyses of MD. 95% CIs around heterogeneity estimates and random-effects prediction intervals could not be calculated for meta-analyses with <3 studies.

Comparison	Outcome	*n*	MD (95% CI)	*P*	*τ* ^2^ (95% CI)	*I* ^2^ (95% CI)	Prediction
Aligners vs. fixed appliances	ABO-OGS total post-Tx	2	0.05 (−9.36, 9.46)	.99	38.72	84%	-
	ABO-OGS: alignment/rotations	2	0.01 (−1.83, 1.84)	.99	1.41	80%	-
	ABO-OGS: marginal ridges	2	0.64 (−0.07, 1.34)	.07	0.14	53%	-
	ABO-OGS: buccolingual inclination	2	0.35 (−0.17, 0.80)	.19	0.03	17%	-
	ABO-OGS: overjet	2	−0.41 (−2.09, 1.28)	.64	0.92	59%	-
	ABO-OGS: occlusal contacts	2	0.58 (−2.50, 3.67)	.71	4.70	95%	-
	ABO-OGS: occlusal relationships	2	0.48 (−0.57, 1.54)	.37	0	0%	-
	ABO-OGS: interproximal contacts	2	0.06 (−0.36, 0.49)	.76	0.05	16%	-
	ABO-OGS: root angulation	2	−0.11 (−1.90, 1.69)	.91	1.53	92%	-
	Tx duration	2	−0.22 (−5.44, 5.00)	.93	11.08	78%	-
CAD/CAM vs. prefabricated brackets	PAR post-Tx	2	−0.15 (−0.78, 0.48)	.64	0	0%	-
	Tx duration	2	−1.43 (−5.42, 2.55)	.48	7.83	95%	-
Skeletal vs. conventional anchorage	PAR post-Tx	2	−1.36 (−5.29, 2.57)	.50	6.38	80%	-
	PAR reduction	2	1.09 (−3.56, 5.73)	.65	0	0%	-
	Tx duration	2	−0.96 (−4.98, 3.07)	.64	0	0%	-
SL vs. conventionally-ligated brackets	PAR postTx	3	0.23 (−0.92, 1.38)	.70	0.49 (0, > 100)	51% (0%, 86%)	−11.39, 11.85
	% PAR reduction	3	−0.42 (−2.04, 1.20)	.62	0 (0, > 100)	0% (0%, 90%)	−10.92, 10.09
	Tx duration	4	−0.08 (−2.36, 2.21)	.95	3.18 (0, 79.10)	58% (0%, 86%)	−9.24, 9.09

ABO, American Board of Orthodontics; CAD/CAM, computer-assisted designed/manufactured; CI, confidence interval; MD, mean difference; OGS, objective grading system; PAR, peer assessment rating; SL, self-ligating; Tx, treatment.

**Figure 2.**
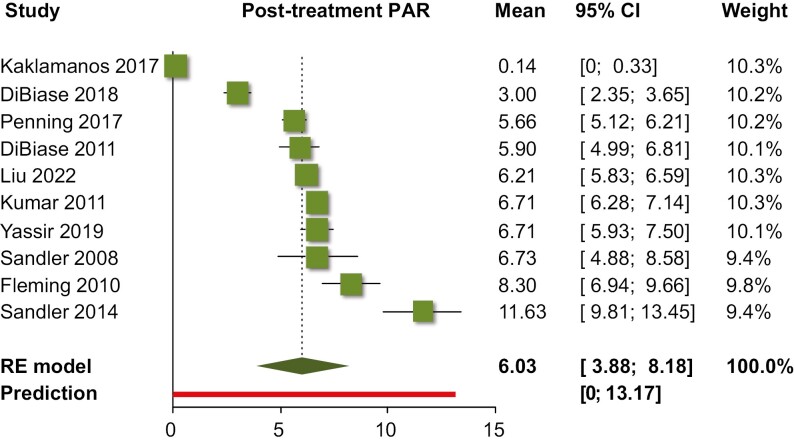
Forest plot for single-group meta-analysis of average posttreatment PAR score. CI, confidence interval; PAR, peer assessment rating; RE, random-effects model.

**Figure 3.**
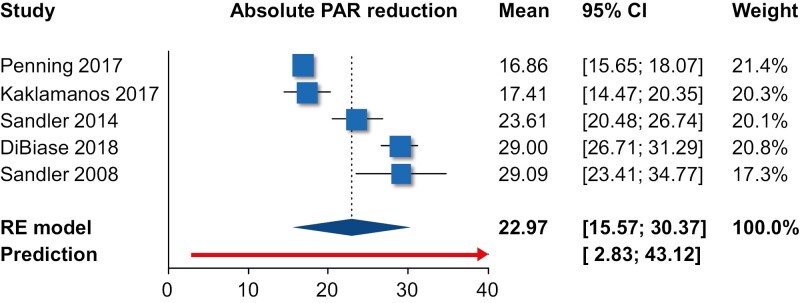
Forest plot for single-group meta-analysis of average absolute PAR reduction. CI, confidence interval; PAR, peer assessment rating; RE, random-effects model.

**Figure 4.**
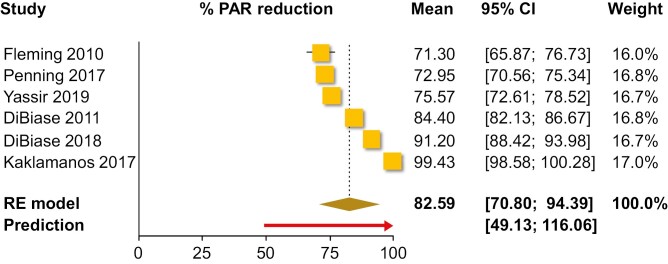
Forest plot for single-group meta-analysis of average % PAR reduction. CI, confidence interval; PAR, peer assessment rating; RE, random-effects model.

**Figure 5.**
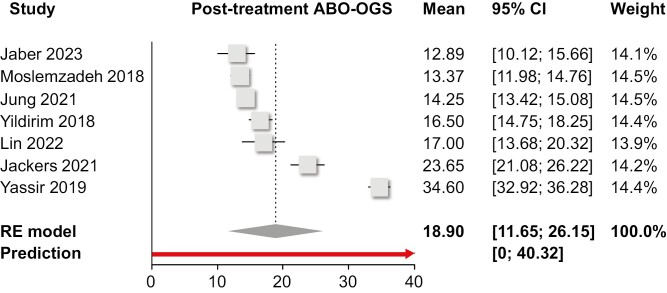
Forest plot for single-group meta-analysis of average posttreatment ABO-OGS score. ABO, American Board of Orthodontics; CI, confidence interval; OGS, objective grading system; RE, random-effects model.

**Figure 6.**
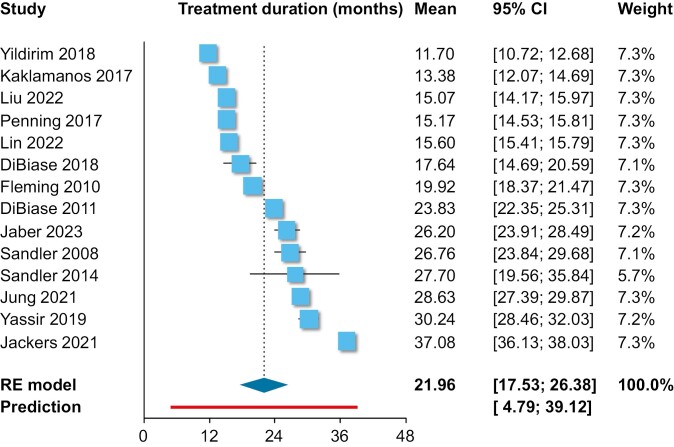
Forest plot for single-group meta-analysis of average treatment duration. CI, confidence interval; PAR, peer assessment rating; RE, random-effects model.

Single-group meta-analysis of averages (Table 2) indicated that as far as the PAR index was concerned, orthodontic treatment resulted in an average post-treatment PAR of 6.0 points (10 trials; 95% CI 3.9–8.2 points; Fig. 2), with an absolute treatment-related PAR reduction of 23.0 points (five trials; 95% CI 15.6–30.4 points; Fig. 3) and a % treatment-related PAR reduction of 82.6% (six trials; 95% CI 70.8%–94.4%; Figure 4). Using the ABO-OGS tool, the pooled average post-treatment score was 18.9 points (7 trials; 95% CI 11.7–26.2 points; Fig. 5), while the most problematic ABO-OGS components were in decreasing order: occlusal contacts (3.7 points), alignment/rotations (3.3 points), buccolingual inclinations (3.2 points), marginal ridges (2.7 points), occlusal relationships (2.2 points), overjet (2.0 points), root angulation (1.3 point), and interproximal contacts (0.2 point). Finally, the pooled average treatment duration was 22.0 months (14 studies; 95% CI 17.5–26.4 months; Fig. 6). However, very high heterogeneity (*I*^2^ > 80%) was observed in all instances, and therefore the 95% CIs might be more informative than the pooled point estimates since they give the distribution of average effects across included studies.

Pairwise (two-group) meta-analyses of MDs (Table 3) and results of single trials (Supplementary Appendix 7) covered four separate subjects: (i) 0.018″-slot versus 0.022″-slot brackets; (ii) CAD/CAM versus prefabricated brackets; (iii) indirectly versus directly bonded brackets; (iv) self-ligating versus conventionally-ligated brackets; (v) skeletal anchorage reinforcement versus conventional anchorage; (vi) vibrational adjuncts to fixed appliances versus no adjuncts; and (vii) orthodontic aligners versus fixed appliances (Table 3).

Results of a single trial found no significant differences between 0.018″-slot and 0.022″-slot brackets in terms of post-treatment PAR, % PAR reduction, post-treatment ABO-OGS, and treatment duration (*P* > .05 in all instances).

Meta-analysis of two trials found no statistically significant difference between Computer-Assisted Designed/Manufactured (CAD/CAM) and pre-fabricated brackets in terms of post-treatment PAR or treatment duration (*P* > .05 in all instances). Data from single trials found no difference in absolute or relative PAR reductions (*P* > .05 in all instances) between CAD/CAM and pre-fabricated brackets but found that the former were associated with improved occlusal contacts (MD −3.3 points; 95% CI −6.0 to −0.6 points; *P* = .01).

Results from a single trial found that indirectly bonded brackets were associated with improved occlusal outcomes overall (MD −5.0 points; 95% CI −8.0 to −2.0 points; *P* = .001) compared to directly bonded brackets, which was mostly due to improved marginal ridges (MD −2.0 points; 95% CI −3.9 to −0.1 points; *P* = 0.03). However, there was no difference in treatment duration between indirectly and directly bonded brackets (*P* = .55).

Meta-analysis of two trials found no statistically significant differences between fixed appliance treatment reinforced with skeletal anchorage or with conventional anchorage means in terms of post-treatment PAR, absolute PAR reduction, or treatment duration (*P* > .05 in all instances).

Meta-analysis of two trials found no significant difference between self-ligating and conventional brackets in terms of post-treatment PAR, % PAR reduction, or treatment duration (*P* > .05 in all instances). On the other hand, results from a single trial indicated that self-ligating brackets were associated with worse overall occlusal outcomes than conventional brackets (MD 2.7 points; 95% CI 1.1–4.3 points; *P* = .001), which was mostly due to worse buccolingual inclination (MD 1.5 point; 95% CI 0.8–2.2 points) and to a much lesser degree to worse root angulation (MD 0.3 point; 95% CI 0–0.6 point; *P* = .02).

A single trial found no significant beneficial effect from vibrational adjunct to fixed appliance treatment in terms of post-treatment PAR, absolute/relative PAR reduction, and treatment duration (*P* > .05 in all instances).

Finally, meta-analysis of two trials found no significant difference in the occlusal outcome or treatment duration with ABO-OGS between treatment with aligners or fixed appliances (*P* > .05 in all instances).

### Additional analyses

Meta-regressions on single-group meta-analyses with at least five trials (Supplementary Appendix 8) showed that post-treatment PAR scores were significantly associated with treatment duration, so that every additional month of treatment was associated with +0.3 PAR point (95% CI 0–0.7 point). Additionally, greater absolute PAR reductions were seen for patients with greater initial PAR scores (+0.6 point reduction for each additional baseline PAR point; 95% CI 0–1.2 points). Patient sex was associated with % PAR reduction, with a 10% increase in male patients being associated with +6.9% PAR reduction (95% CI −0.5 to +14.3 points). Finally, each additional pre-treatment PAR point was associated with an increase by 0.6 month in treatment duration (95% CI 0.1–1.1 month).

Subgroup analysis according to the inclusion of tooth extractions in the treatment plan (Table 4) indicated that extraction cases showed significantly greater absolute PAR reduction than non-extraction cases (29.0 versus 16.9 points, respectively). Reversely, extraction cases had better occlusal outcomes through ABO-OGS than non-extraction cases (12.9 versus 16.6 points, respectively). Finally, extraction treatment took significantly longer than non-extraction treatment on average (20.6 versus 14.2 months, respectively).

**Table 4.** Subgroup analyses of single-group meta-analyses of averages.

		Extraction cases		Mixed cases		Non-Extraction cases	*P*_subgroup_
**Outcome**	***n***	**Mean (95% CI)**	***n***	**Mean (95% CI)**	***n***	**Mean (95% CI)**	
PAR post-Tx	3	5.69 (0, 12.27)	3	8.31 (1.33, 15.29)	3	4.00 (0, 12.35)	0.21
PAR reduction	1	29.00 (26.71, 31.29)	2	25.82 (0, 59.96)	2	16.94 (14.48, 19.40)	<0.001
% PAR reduction	3	82.51 (57.60, 100.00)	1	75.57 (72.61, 78.52)	2	86.22 (0, 100.00)	0.38
ABO-OGS total post-Tx	1	12.89 (10.12, 15.66)	2	24.41 (0, >100.00)	2	16.61 (13.99, 19.23)	0.02
Tx duration	4	21.96 (15.93, 27.99)	4	28.79 (26.51, 31.07)	5	14.24 (12.22, 16.25)	<0.001

ABO, American Board of Orthodontics; OGS, objective grading system; PAR, peer assessment rating; Tx, treatment.

Contour-enhanced funnel plots to assess reporting biases for single-group meta-analyses with ≥10 studies are shown in Supplementary Appendices 9 and 10. Funnel plot asymmetry was confirmed for post-treatment PAR scores (Thompson’s test *P* = .02), which was deemed to be due to small-study effects (effect overestimation from small/imprecise studies). This was confirmed by sensitivity analysis (Supplementary Appendix 11) that divided available trials into two subsets of precise or imprecise studies (based on their standard errors) and found that precise studies found significantly smaller post-treatment PAR scores than imprecise studies (4.3 versus 7.8 points, respectively).

The quality of evidence for all pairwise (two-group) meta-analyses was moderate in three instances due to the inclusion of trials with a high risk of bias and low in the remaining seven instances due to additional imprecision risk from the inclusion of a limited number of patients (Table 5). Sensitivity analysis by risk of bias (essentially including only one of the two trials in each instance that was at low risk of bias) showed no large differences to the main analysis (Supplementary Appendix 12). Changed findings included (i) significantly worse occlusal outcomes through ABO-OGS for aligners compared to fixed appliances and (ii) significantly better occlusal outcomes through posttreatment PAR with skeletal compared to conventional anchorage.

**Table 5.** Summary of findings table for pairwise (two-group) meta-analyses according to the GRADE approach.

	Anticipated absolute effects (95% CI)			
**Outcome** **Studies (patients)**	**Reference** ^a^	**Difference in experimental**	**Quality of the** **evidence (GRADE)** ^b^	**What happens with experimental**
	Fixed appliances	Aligners		
ABO-OGS total post-Tx two studies (91 patients)	14.9 points	0.1 point more (9.4 less to 9.5 more)	⊕⊕○○ low^c,d^ due to bias, imprecision	No difference in finishing quality
Tx duration two studies (91 patients)	20.9 months	0.2 months less (5.4 less to 5.0 more)	⊕⊕○○ low^c,d^ due to bias, imprecision	No difference in Tx duration
	Prefabricated brackets	CAD/CAM brackets		
PAR post-Tx two studies (245 patients)	6.1 points	0.2 point less (0.8 less to 0.5 more)	⊕⊕⊕○ moderate^c^ due to bias	No difference in post-Tx occlusal outcome
Tx duration two studies (198 patients)	15.7 months	1.4 month less (5.4 less to 2.6 more)	⊕⊕⊕○ moderate^c^ due to bias	No difference in Tx duration
	Conventional anchorage	Skeletal anchorage		
PAR post-Tx two studies (119 patients)	9.2 points	1.4 point less (5.3 less to 2.6 more)	⊕⊕○○ low^c,d^ due to bias, imprecision	No difference in post-Tx occlusal outcome
Tx-related PAR reduction two studies (119 patients)	25.8 points	1.1 point more (3.6 less to 5.7 more)	⊕⊕○○ low^c,d^ due to bias, imprecision	No difference in Tx-related occlusal outcome improvement
Tx duration two studies (119 patients)	26.9 months	1.0 month less (5.0 less to 3.1 more)	⊕⊕○○ low^c,d^ due to bias, imprecision	No difference in Tx duration
	Conventional brackets	Self-ligating brackets		
PAR post-Tx three studies (124 patients)	4.4 points	0.2 point more (0.9 less to 1.4 more)	⊕⊕○○ low^c,d^ due to bias, imprecision	No difference in post-Tx occlusal outcome
Tx-related % PAR reduction three studies (124 patients)	86.2%	0.4% less (2.0 less to 1.2 more)	⊕⊕○○ low^c,d^ due to bias, imprecision	No difference in Tx-related %occlusal outcome improvement
Tx duration four studies (258 patients)	21.3 months	0.1 month less (2.4 less to 2.2 more)	⊕⊕⊕○ moderate^c^ due to bias	No difference in Tx duration

Intervention: comprehensive orthodontic treatment with fixed appliances; Population: underage or adult patients with any kind of malocclusion in need of treatment; Setting: university clinics, hospitals, and private practices (Belgium, China, Great Britain, Greece, South Korea, Syria, the Netherlands, and United States of America).

^a^Response in the control group is based on random-effects meta-analysis of the control response.

^b^Starts from “high”. ^c^Downgraded by one level for bias due to the risk of bias of included study. ^d^ Downgraded by one level for imprecision due to the inclusion of a small number of patients overall.CI, confidence interval; GRADE, grading of recommendations assessment, development and evaluation; Tx, treatment.

## Discussion

This systematic review critically appraised evidence from randomized trials assessing objective occlusal outcome with either the PAR or the ABO-OGS index after comprehensive fixed appliance treatment on adolescent or adult patients. Data from twenty small- to moderately-sized trials from twelve countries covering 1470 patients indicated that orthodontic treatment with fixed appliances is effective and results on average in final PAR scores of 6.0 points, average absolute PAR reductions of 82.6%, and absolute ABO-OGS scores of 18.9 points. However, very high between-study heterogeneity was seen for both indices, and even though some of it could be explained by patient- or treatment-related characteristics, potential issues pertaining to the use of these indices remain.

Part of the observed heterogeneity between studies could be explained by differences in whether tooth extractions were performed or not in the samples of the included studies. Subgroup analyses (Table 5) indicated that greater absolute PAR reductions were seen for extraction patients compared to non-extraction patients (29.0 versus 16.9 points). However, it should be noted here that absolute PAR reductions are directly associated with the baseline PAR severity. Within the included studies, the pooled pretreatment PAR score was 29.7 points (10 studies; 95% CI 25.2–34.2 points), and extraction patients had significantly more severe pre-treatment PARs than non-extraction patients (34.8 versus 20.5 points; *P* < .001). This could explain the observed differences in absolute PAR reductions, while the % PAR reductions were similar for extraction and non-extraction patients (82.5% versus 86.2%; Table 5). On the other hand, the ABO-OGS index showed that extraction cases had significantly better occlusal outcomes than non-extraction cases (12.9 versus 16.6 points), and this index is not directly associated with pre-treatment PAR scores. Improved occlusal outcomes with extraction treatment at debond have previously been reported from observational studies [23, 24, 44], while extraction cases have also been reported to show less relapse post-debond compared to non-extraction cases [18, 45]. However, it remains to be seen whether this is due to extraction-related factors or simply due to the on average improved occlusal outcome of these patients.

Bracket slot size choice was not found to considerably affect the occlusal outcome of fixed appliance orthodontic treatment. A single trial, divided in four separate papers [33, 34, 46, 47], found no differences between 0.018″-slot and 0.022″-slot brackets in terms of overall PAR changes, total ABO-OGS, or treatment duration, even though 0.018″-slot patients showed marginally better marginal ridges than 0.022″-slot patients through the corresponding ABO-OGS component (Supplementary Appendix 7). Published reports of this trial not included in this review [46, 47] also reported no differences in anchorage loss, external apical root resorption, or pain perception. Other studies have provided additional small benefits for 0.018″-slot systems in terms of improved canine retraction post-extraction [48] or easier torque application [49, 50]—the clinical relevance of whom however remains unclear.

Indirect bonding of orthodontic brackets and its impact on the treatment’s occlusal outcome was assessed only from a single trial [51], which found overall better total ABO-OGS scores—mostly due to improved marginal ridges (Supplementary Appendix 7). This might be due to improved accuracy of bracket positioning that helps align the marginal ridges during the leveling and alignment phase. A cost-minimization analysis showed that, even though indirect bonding is associated with reduced chair time, when the time needed to digitally place the brackets on the teeth is added, indirect bonding is associated with longer total bonding times, while increased bond failure contributed to overall greater costs than direct bonding [52]. Evidence on the effect of indirect bonding on tooth demineralization is conflicting [53, 54] and it seems that removal of excess resin remnants around the brackets is important to keep the demineralization risk low.

The use of custom-made brackets for each patient using CAD/CAM technologies had only a minor effect on the occlusal outcomes of orthodontic treatment. Three included trials compared a CAD/CAM self-ligating bracket system (Insignia, Ormco, Calif) to a prefabricated self-ligating bracket system (Damon Q, Ormco, Calif) and found no significant differences in overall PAR or ABO-OGS, while only minimally improved occlusal contacts were found through the corresponding ABO-OGS component. It is important, however, here to stress that the CAD/CAM bracket used is bonded indirectly, while the compared prefabricated bracket was bonded indirectly in only one of the three trials [55] and directly in the other two [36, 56]. On the other hand, the one trial using indirect bonded for both brackets was only partly randomized, since the CAD/CAM data originated from a trial arm of a previous randomized trial, while the prefabricated data originated from consecutive patients. Therefore, potential bias exists and current evidence on the clinical performance of these brackets is problematic.

Self-ligating orthodontic brackets were similarly found to have a small effect on the occlusal outcome of fixed appliance treatment with no overall difference to conventionally-ligated brackets in terms of PAR scores or treatment duration. A single recent quasi-randomized study reported that self-ligating brackets were associated with slightly worse occlusal outcome through total ABO-OGS scores [57], and this was mostly due to worse buccolingual inclinations and root parallelism. However, this was a single study with a high risk of bias and does not provide conclusive evidence. Self-ligating brackets have been shown to perform similarly to conventional brackets for the most part [58]. Minor benefits of self-ligating brackets include slightly quicker archwire insertion/removal and a clinically irrelevant reduction in alignment duration, while minor drawbacks include increased pain during insertion/removal of a fully-sized working archwire [59].

Anchorage reinforcement with the use of temporary anchorage devices (miniscrew implants, palatal implants, or mini-plates) was not associated with overall improved occlusal outcomes through PAR or reduced treatment duration. Use of temporary anchorage devices has been proven to lead to less anchorage loss than other anchorage reinforcement means (headgear, transpalatal arches, Nance buttons, or differential forces) [60, 61], but this does not seem to necessarily translate to improved occlusal outcomes or efficiency.

The use of vibrational adjuncts was not associated with improved PAR scores or reduced treatment duration compared to fixed appliances alone. Supplemental vibration has similarly not been proven to have any clinically relevant benefits in terms of tooth alignment, space closure, pain experience, and external apical root resorption [62]. Therefore, its use cannot be justified.

Orthodontic aligners were compared to fixed appliances in two randomized trials [40, 41] that overall found no significant differences in ABO-OGS scores or treatment duration. This agrees partly with a previous systematic review of randomized or matched for baseline malocclusion severity non-randomized studies [25], which found no difference in treatment duration but worse occlusal outcome with ABO-OGS for aligners. However, one of the two included trials [40] had considerable baseline imbalances in the malocclusion severity (assessed through the ABO-DI) between the aligner and the fixed appliance group, which could be either due to improper/ineffective randomization or due to chance but should probably be accounted for in the statistical analysis [63]. Unfortunately, attempts to contact the trial’s authors to retrieve the trial’s data were unsuccessful (Supplementary Appendix 4), and therefore, potential biases could not be assessed. Sensitivity analysis by removing this high-risk trial (essentially relaying the results of the other trial [41]) indicated that aligners were associated with worse occlusal outcome using ABO-OGS, which was mostly due to worse occlusal contacts and less due to worse root angulation, buccolingual inclination, and alignment. This agrees with data from observational studies reporting issues with adequately controlling root angulation, buccolingual inclination, and alignment [64–66] and raises questions about the efficacy of orthodontic aligners on realizing planned tooth movements.

As far as the use of occlusal outcome indices is concerned, the PAR index can usually be reported in a paper either as (i) absolute post-treatment PAR value, (ii) absolute treatment-related (pre-treatment minus post-treatment) PAR reduction, or (iii) % treatment-related PAR reduction. It is unclear which of the three options is preferential and if more than one should be reported. The absolute post-treatment PAR is essentially a measure of “residual malocclusion” and, as such, does not directly reflect treatment efficacy. On the other hand, absolute PAR reduction is directly associated to baseline PAR severity and can be expected to be greater in more severe cases (with higher pre-treatment PAR scores). Therefore, it might be appropriate to jointly report both absolute and relative measures of occlusal outcome to aid in their interpretation.

Finally, the fact that so large between-study heterogeneity was seen in the occlusal outcomes across trials raises questions about the consistency with which each index was used. In other words, since the measured material (i.e. the patients’ plaster or digital models at debond) is not available to validate the measured occlusal outcome, it remains unclear whether the same diligence or strictness was used across all research teams. Currently, manual scoring of the PAR index using either the physical models (be it plaster or three-dimensionally [3D] printed ones) or digital models has been shown to be equally reliable [67, 68]. On the other hand, Nguyen in her thesis [69] reported that manual scoring of digital models has been shown to give slightly higher ABO-OGS scores than obtained by manual scoring of plaster models, which was interpreted by the author to mean that digital ABO-OGS scoring is not reliable. However, digitization of the scoring process for such occlusal indices is the first step in enabling open research practices with adequate reliability and reproducibility. Ideally, such scorings could be performed in an automated fashion that ensures the same measurements are taken from different people, at different times, and in different conditions. Automated or semi-automated scoring methods for the PAR index exist, can be applied either directly during intraoral scanning or on the digital scan files [70], and have been reported to have good validity and reliability [70, 71], which was better than the manual scoring of either plaster or digital models [70] and eliminated variation [71]. On the other hand, automated scoring methods for digital models gave significantly higher overall ABO-OGS scores than manual scoring of physical 3D printed models, which affected alignment, rotations, and occlusal contacts [72]. Further research is currently needed to evaluate whether automated scoring protocols for both indices perform adequately, but in time, eliminating the potential errors from manual scoring will be beneficial in increasing the consistency, reliability, and reproducibility of these methods.

### Limitations

The present systematic review has several limitations that should be discussed. Issues with the randomization process were found for several included trials (Supplementary Appendix 5), and this is associated with bias [73]. However, sensitivity analysis was undertaken (Supplementary Appendix 12) to include only trials with low risk of bias, and its results were consistent with the main analysis. Additionally, very high (>75%) between-study heterogeneity was observed for all single-group meta-analyses of averages. Even though some heterogeneity was explained by the meta-regression/subgroup analyses (Table 5; Supplementary Appendix 8), focus should not be placed solely on the pooled average estimates but also on the random-effects predictions, which incorporate observed heterogeneity and provide a range of outcomes in a future study. Furthermore, only a few corresponding authors responded to our requests for additional or raw data (Supplementary Appendix 4), and this affected the volume and extent of the performed statistical analyses [74]. Most trials included small samples (with only four trials [36, 47, 56, 57] having >30 patients per experimental group), and this could influence the meta-analytical estimates [75, 76]. Moreover, not all trials had a pre-registered protocol, and none presented an a priori statistical analysis plan, which impairs their transparency and might be associated with bias [77, 78]. Finally, even though the review’s main focus is on fixed appliances, some trials also included removable appliances like aligners and retainers, and compliance-related factors (including, among others, age, sex, and motivation) might influence their outcomes [79, 80].

## Conclusions

Randomized trials on comprehensive orthodontic treatment of underage or adult patients with fixed appliances give very heterogeneous data on the occlusal outcome measured with the PAR or ABO-OGS index. Considerable differences were seen between extraction and non-extraction treatment, with the former being associated with better occlusal outcomes but longer treatment duration. No evidence for improved occlusal outcomes was seen with the use of 0.018”-slot or 0.022”-slot brackets, customized brackets, self-ligating brackets, temporary anchorage devices, vibrational adjuncts, or aligners. However, the consistency to which these occlusal indices are applied from different researchers remains questionable, and future efforts should focus on developing standardized methods to reliably measure the occlusal outcome of orthodontic treatment.

## Supplementary Material

cjae060_suppl_Supplementary_Appendix

## Data Availability

The study’s dataset is openly available through Zenodo (doi: 10.5281/zenodo.10839826).

## Registration

PROSPERO (CRD42024525088).
