# Supplementary material for: Occlusal outcome of orthodontic treatment: a systematic review with meta-analyses of randomized trials
Source: Eur J Orthod. 2024 Nov 28;46(6):cjae060. doi: 10.1093/ejo/cjae060 (PMC11602743; doi:10.1093/ejo/cjae060)
Supplement: cjae060_suppl_Supplementary_Appendix [file cjae060_suppl_supplementary_appendix.docx]

**Occlusal outcome of orthodontic treatment: a systematic review with meta-analyses of randomized trials**

**SUPPLEMENTARY MATERIAL**

**Appendix 1.** Additional review details.

- Whenever needed, appropriate data transformations were performed to prepare data for synthesis. This includes pooling similar study-arms to provide a common estimate for all fixed appliances of a study and converting non-parametric to parametric data.
- For the sensitivity analysis according to study precision, the standard error of the estimate was used to categorize studies as precise (5/10 studies with the smallest standard error) or imprecise (the remaining 5/10 studies with the largest standard error).
- All estimates for PAR and ABO-OGS scores were truncated at zero.
- For the results of single trials, clinical relevance was judged as effects being larger than half a standard deviation of the response in the control group.

Deviations from protocol

- We changed the test used for funnel plot asymmetry from the Egger’s test to the Thompson test, which has been shown to perform better.

# 1

**Appendix 2.** Literature searches conducted in each database with retrieved hits (last searchdate March 5^th^, 2024).

| **Database** | **Search** | **Filters** | **Hits** |
| --- | --- | --- | --- |
| MEDLINE (via Pubmed) | orthodon* AND ("Peer Assessment Rating" OR "PAR index" OR "PAR score" OR "PAR tool" OR "American Board of Orthodontics" OR "ABO index" OR "ABO score" OR "ABO tool" OR "objective grading" OR "occlusal  outcome" OR "occlusal quality" OR "finishing quality") AND (random* OR blind* OR trial OR CONSORT) | Human | 179 |
| Web of Science | Same as Pubmed | Dentistry Oral Surgery | 160 |
| Scopus | Same as Pubmed | Dentistry | 115 |
| Embase | Same as Pubmed |  | 20 |
| Cochrane Central Register of Controlled Trials | Same as Pubmed |  | 71 |
| Cochrane Database of Systematic Reviews | Same as Pubmed |  | 2 |
| Virtual Health Library | Same as Pubmed |  | 10 |
| **Sum (with overlap)** | | | **557** |
| **Sum (without overlap)** | | | **265** |

# 2

**Appendix 3.** List of hits identified from the literature search and their inclusion / exclusion status (with reasons).

| **Nr** | **Paper** | **Stat** |
| --- | --- | --- |
| 1 | Ackerman M. Evidence-based orthodontics for the 21st century. J Am Dent Assoc. 2004;135(2):162-7; quiz 227-8. | Exclusion by title |
| 2 | Alsaeed S, Aljarallah S, Alarjani A, Alghunaim G, Alanizy A. Dental malpractice lawsuit cases in Saudi Arabia: A national study. Saudi Dental Journal. 2022;34(8):763- 71. | Exclusion by title |
| 3 | Jayakumar P, FelsyPremila G, Muthu MS, Kirubakaran R, Panchanadikar N, Al-Qassar SS. Bite force of children and adolescents: a systematic review and meta-  analysis. J Clin Pediatr Dent. 2023;47(3):39-53. | Exclusion by title |
| 4 | Bagattoni S. Genetic/chromosomal syndromes and orthodontics: treatment efficacy and literature review. Dental Cadmos. 2023;91(2):104-15. | Exclusion by abstract |
| 5 | Bailey L. Accuracy of CBCT-generated cephalometric radiographs for ABO analysis. 2013. | Exclusion by abstract |
| 6 | Bakhsh Z. A new methodological approach to selecting and weighting criteria for the Objective Grading System. 2008. | Exclusion by abstract |
| 7 | Barnard MA. Bolton discrepancy in a selected Canadian population. 2008. | Exclusion by abstract |
| 8 | Bollen AM. Herbst appliance more effective than twin-block appliance in treating adolescent Class II division I malocclusions. Journal of Evidence-Based Dental Practice. 2004;4(3):216-7. | Exclusion by abstract |
| 9 | Drew J. Measuring a Sample of Orthodontic Models/Panographs at LLUSD Using the CR-Eval. 2018. | Exclusion by abstract |
| 10 | Dykhouse VJ, Moffitt AH, Grubb JE, Greco PM, English JD, Briss BS, et al. A report of the ABO Resident Clinical Outcome Study (the pilot study). Am J Orthod Dentofacial Orthop. 2006;130(5):656-61. | Exclusion by abstract |
| 11 | Fricton JR, Ouyang W, Nixdorf DR, Schiffman EL, Velly AM, Look JO. Critical appraisal of methods used in randomized controlled trials of treatments for  temporomandibular disorders. J Orofac Pain. 2010;24(2):139-51. | Exclusion by abstract |
| 12 | Hall JF, Sohn W, McNamara JA. Why do dentists refer to specific orthodontists? Angle Orthod. 2009;79(1):5-11. | Exclusion by abstract |
| 13 | Jacox LA. Orthognathic speech pathology: Understanding how jaw disharmonies and their surgical correction influence speech. 2021. | Exclusion by abstract |
| 14 | Josell SD. Interceptive orthodontic treatment may be effective in reducing the severity of developing malocclusion. Journal of Evidence-Based Dental Practice. 2011;11(1):31-2. | Exclusion by abstract |
| 15 | Jung MH. Factors influencing treatment efficiency. Angle Orthod. 2021;91(1):1-8. | Exclusion by abstract |
| 16 | Kalha A. Early treatment with the twin-block appliance is effective in reducing overjet and severity of malocclusion: Is the twin-block orthodontic appliance effective in early treatment of developing class II division 1 malocclusion? Evidence-Based Dentistry. 2004;5(4):102-3. | Exclusion by abstract |
| 17 | Kalha AS. Early orthodontic treatment reduced incisal trauma in children with class II malocclusions. Evid Based Dent. 2014;15(1):18-20. | Exclusion by abstract |
| 18 | Kalha AS. Hawley or vacuum-formed retainers following orthodontic treatment? Evid Based Dent. 2014;15(4):110-1. | Exclusion by abstract |
| 19 | Kassam SK, Stoops FR. Are clear aligners as effective as conventional fixed appliances? Evid Based Dent. 2020;21(1):30-1. | Exclusion by abstract |
| 20 | Khamis N, Monari E, Taddei M, Tagariello T, Ghidoni R, Fitzgibbon R, et al. Genetic/chromosomal syndromes and orthodontics: treatment efficacy and literature review. Dental Cadmos. 2023;91(2). | Exclusion by abstract |
| 21 | King G. Early orthodontic growth modification treatment for Class II patients may provide better skeletal and dental outcomes after subsequent comprehensive permanent dentition orthodontic treatment with less need for complex interventions and greater efficiency. Journal of Evidence-Based Dental Practice. 2011;11(1):49-  51. | Exclusion by abstract |
| 22 | Langella F, Fusini F, Rossi G, Villafañe JH, Migliaccio N, Donzelli S, et al. Spinal deformity and malocclusion association is not supported by high-quality studies: results from a systematic review of the literature. Eur Spine J. 2019;28(7):1638-51. | Exclusion by abstract |
| 23 | Liu S, Oh H, Chambers DW, Baumrind S, Xu T. Validity of the American Board of Orthodontics Discrepancy Index and the Peer Assessment Rating Index for comprehensive evaluation of malocclusion severity. Orthod Craniofac Res. 2017;20(3):140-5. | Exclusion by abstract |
| 24 | Luqmani S, Jones A, Andiappan M, Cobourne MT. A comparison of conventional vs automated digital Peer Assessment Rating scoring using the Carestream 3600  scanner and CS Model+ software system: A randomized controlled trial. Am J Orthod Dentofacial Orthop. 2020;157(2):148-55.e1. | Exclusion by abstract |
| 25 | Madurantakam P. Fixed or removable function appliances for Class II malocclusions. Evid Based Dent. 2016;17(2):52-3. | Exclusion by abstract |
| 26 | Miethke RR, Wronski C. What can be achieved with removable orthodontic appliances? J Orofac Orthop. 2009;70(3):185-99. | Exclusion by abstract |
| 27 | Mir CF. One-phase or two-phase orthodontic treatment? Evid Based Dent. 2016;17(4):107-8. | Exclusion by abstract |
| 28 | Noble J, Hechter FJ, Karaiskos NE, Lekic N, Wiltshire WA. Future practice plans of orthodontic residents in the United States. Am J Orthod Dentofacial Orthop 2009;135(3):357-60. | Exclusion by abstract |
| 29 | Papakostopoulou M, Hurst D. Customised fixed appliance systems and treatment duration. Evid Based Dent. 2018;19(2):50. | Exclusion by abstract |
| 30 | Radeke J, von der Wense C, Lapatki BG. Comparison of orthodontic measurements on dental plaster casts and 3D scans. Journal of Orofacial Orthopedics- Fortschritte Der Kieferorthopadie. 2014;75(4):264-74. | Exclusion by abstract |
| 31 | Ren Y. Very few indications justify early treatment for severe class II malocclusions: Are there superior results when treatment of class II malocclusions is started before adolescence and followed by a second phase of treatment in the earlypermanent dentition, compared with a single-phase treatment that is delayed until adolescence? Evidence-Based Dentistry. 2004;5(4):100-1. | Exclusion by abstract |
| 32 | Rheude B, Sadowsky PL, Ferriera A, Jacobson A. An evaluation of the use of digital study models in orthodontic diagnosis and treatment planning. Angle Orthod. 2005;75(3):300-4. | Exclusion by abstract |
| 33 | Rossini G, Parrini S, Castroflorio T, Deregibus A, Debernardi CL. Diagnostic accuracy and measurement sensitivity of digital models for orthodontic purposes: A systematic review. Am J Orthod Dentofacial Orthop. 2016;149(2):161-70. | Exclusion by abstract |
| 34 | Sandoval P, Bizcar B. Beneficios de la Implementación de Ortodoncia Interceptiva en la Clínica Infantil Dental Clinic. International journal of odontostomatology. 2013;7(2):253-65. | Exclusion by abstract |
| 35 | Stevens DR. Clinical use of virtual study models versus traditional plaster study models for orthodontic treatment diagnosis. 2004. | Exclusion by abstract |
| 36 | Veenema AC, Katsaros C, Boxum SC, Bronkhorst EM, Kuijpers-Jagtman AM. Index of Complexity, Outcome and Need scored on plaster and digital models. European Journal of Orthodontics. 2009;31(3):281-6. | Exclusion by abstract |
| 37 | Vig K. One- or two-phase orthodontic treatment for Class II malocclusion does not change the occlusal outcome. Journal of Evidence-Based Dental Practice. 2004;4(2):142-3. | Exclusion by abstract |
| 38 | Yount KA. Appliance design and application. Gen Dent. 2012;60(6):e359-77; quiz p. e78-9. | Exclusion by abstract |
| 39 | Zahavi S, Caminiti M. IN-HOUSE THREE-DIMENSIONALLY PRINTED SPLINT FABRICATION FOR ORTHOGNATHIC SURGERY: ARE THESE INFERIOR TO  INDUSTRY STANDARDS? International Journal of Oral and Maxillofacial Surgery. 2024;52:188. | Exclusion by abstract |
| 40 | Aksakalli S, Temucin F, Pamukcu A, Ezirganlı S, Kazancioglu HO, Malkoc MA. Effectiveness of two different splints to treat temporomandibular disorders. J Orofac Orthop. 2015;76(4):318-27. | Excluded; not orthodontics- related |
| 41 | Al-Moraissi EA, Wolford LM, Ellis E, 3rd, Neff A. The hierarchy of different treatments for arthrogenous temporomandibular disorders: A network meta-analysis of randomized clinical trials. J Craniomaxillofac Surg. 2020;48(1):9-23. | Excluded; not orthodontics- related |
| 42 | Amat P, Tran Lu YE. [Orofacial myofunctional reeducation assisted by a prefabricated reeducation appliance: a systematic review of the literature]. Orthod Fr. 2023;94(1):131-61. | Excluded; not orthodontics- related |
| 43 | Baraka M, Cevidanes L, Tekeya M, Bakry N, Ruellas A, Botero T, et al. Three-Dimensional Assessment of Radiographic Changes after Indirect Pulp Capping Using Silver Diamine Fluoride with or without Potassium Iodide in Young Permanent Teeth (12-Month RCT). Caries Res. 2023;57(2):177-88. | Excluded; not orthodontics- related |
| 44 | Bartolucci ML, Bortolotti F, Corazza G, Incerti Parenti S, Paganelli C, Alessandri Bonetti G. Effectiveness of different mandibular advancement device designs in obstructive sleep apnoea therapy: A systematic review of randomised controlled trials with meta-analysis. J Oral Rehabil. 2021;48(4):469-86. | Excluded; not orthodontics- related |
| 45 | Bishop B, Verrett R, Girvan T. A randomized crossover study comparing two mandibular repositioning appliances for treatment of obstructive sleep apnea. Sleep  Breath. 2014;18(1):125-31. | Excluded; not orthodontics-  related |
| 46 | Bortolotti F, Corazza G, Bartolucci ML, Incerti Parenti S, Paganelli C, Alessandri-Bonetti G. Dropout and adherence of obstructive sleep apnoea patients to mandibular advancement device therapy: A systematic review of randomised controlled trials with meta-analysis and meta-regression. J Oral Rehabil. 2022;49(5):553-72. | Excluded; not orthodontics- related |
| 47 | Brunetto DP, Moschik CE, Dominguez-Mompell R, Jaria E, Sant'Anna EF, Moon W. Mini-implant assisted rapid palatal expansion (MARPE) effects on adult obstructive sleep apnea (OSA) and quality of life: a multi-center prospective controlled trial. Prog Orthod. 2022;23(1):3. | Excluded; not orthodontics- related |
| 48 | Cheng CW, Ye SY, Chien CH, Chen CJ, Papaspyridakos P, Ko CC. Randomized clinical trial of a conventional and a digital workflow for the fabrication of interim crowns: An evaluation of treatment efficiency, fit, and the effect of clinician experience. J Prosthet Dent. 2021;125(1):73-81. | Excluded; not orthodontics- related |
| 49 | Deane SA, Cistulli PA, Ng AT, Zeng B, Petocz P, Darendeliler MA. Comparison of mandibular advancement splint and tongue stabilizing device in obstructive sleep  apnea: a randomized controlled trial. Sleep. 2009;32(5):648-53. | Excluded; not orthodontics-  related |
| 50 | Dort L, Brant R. A randomized, controlled, crossover study of a noncustomized tongue retaining device for sleep disordered breathing. Sleep Breath. 2008;12(4):369- 73. | Excluded; not orthodontics- related |
| 51 | Ferguson KA. The role of oral appliance therapy in the treatment of obstructive sleep apnea. Clin Chest Med. 2003;24(2):355-64. | Excluded; not orthodontics- related |
| 52 | Fidalgo T, Americano G, Medina D, Athayde G, Letieri ADS, Maia LC. Adhesiveness of bulk-fill composite resin in permanent molars submitted to Streptococcus mutans biofilm. Braz Oral Res. 2019;33:e111. | Excluded; not orthodontics- related |
| 53 | Gesch D, Bernhardt O, Kirbschus A. Association of malocclusion and functional occlusion with temporomandibular disorders (TMD) in adults: a systematic review of population-based studies. Quintessence Int. 2004;35(3):211-21. | Excluded; not orthodontics- related |
| 54 | Hesse D, de Araujo MP, Olegário IC, Innes N, Raggio DP, Bonifácio CC. Atraumatic Restorative Treatment compared to the Hall Technique for occluso-proximal  cavities in primary molars: study protocol for a randomized controlled trial. Trials. 2016;17:169. | Excluded; not orthodontics-  related |
| 55 | Huynh N, Drouin-Gagné L, Gilbert C, Arcache JP, Rompré P, Morency AM, et al. Adherence and efficacy of mandibular advancement splint treatment of sleep- disordered breathing during pregnancy: a pilot study. Sleep Breath. 2023;27(3):869-77. | Excluded; not orthodontics- related |
| 56 | Johal A. Health-related quality of life in patients with sleep-disordered breathing: effect of mandibular advancement appliances. J Prosthet Dent. 2006;96(4):298-302. | Excluded; not orthodontics-  related |
| 57 | Landry ML, Rompré PH, Manzini C, Guitard F, de Grandmont P, Lavigne GJ. Reduction of sleep bruxism using a mandibular advancement device: an experimental controlled study. Int J Prosthodont. 2006;19(6):549-56. | Excluded; not orthodontics- related |
| 58 | Madani AS, Abdollahian E, Khiavi HA, Radvar M, Foroughipour M, Asadpour H, et al. The efficacy of gabapentin versus stabilization splint in management of sleep  bruxism. J Prosthodont. 2013;22(2):126-31. | Excluded; not orthodontics-  related |
| 59 | Marinho VC. Cochrane reviews of randomized trials of fluoride therapies for preventing dental caries. Eur Arch Paediatr Dent. 2009;10(3):183-91. | Excluded; not orthodontics- related |

| 60 | Miotto E, Salvatore Freitas KM, Mori AA, Valarelli FP, Gobbi de Oliveira RC, Oliveira RC. Effect of botulinum toxin on quality of life of patients with chronic myofascial pain. Pain Manag. 2021;11(5):583-93. | Excluded; not orthodontics- related |
| --- | --- | --- |
| 61 | Phillips C, White RP, Jr., Shugars DA, Zhou X. Risk factors associated with prolonged recovery and delayed healing after third molar surgery. J Oral Maxillofac Surg.  2003;61(12):1436-48. | Excluded; not orthodontics-  related |
| 62 | Rose E, Staats R, Virchow C, Jonas IE. A comparative study of two mandibular advancement appliances for the treatment of obstructive sleep apnoea. Eur J Orthod. 2002;24(2):191-8. | Excluded; not orthodontics- related |
| 63 | Satokawa Y, Minami I, Wakabayashi N. Short-term changes in chewing efficiency and subjective evaluation in normal dentate subjects after insertion of oral  appliances with an occlusal flat table. J Oral Rehabil. 2018;45(2):116-25. | Excluded; not orthodontics-  related |
| 64 | Singh PK, Alvi HA, Singh BP, Singh RD, Kant S, Jurel S, et al. Evaluation of various treatment modalities in sleep bruxism. J Prosthet Dent. 2015;114(3):426-31. | Excluded; not orthodontics- related |
| 65 | Smith AM, Battagel JM. Non-apneic snoring and the orthodontist: the effectiveness of mandibular advancement splints. J Orthod. 2004;31(2):115-23. | Excluded; not orthodontics-  related |
| 66 | Song YL, Yap AU. Outcomes of therapeutic TMD interventions on oral health related quality of life: A qualitative systematic review. Quintessence Int. 2018;49(6):487- 96. | Excluded; not orthodontics- related |
| 67 | Stafuzza TC, Vitor LLR, Lourenço Neto N, Rios D, Cruvinel T, Sakai VT, et al. Pulp liner materials in selective caries removal: study protocol for a randomised  controlled trial. BMJ Open. 2021;11(1):e029612. | Excluded; not orthodontics-  related |
| 68 | Vollú AL, Rodrigues GF, Rougemount Teixeira RV, Cruz LR, Dos Santos Massa G, de Lima Moreira JP, et al. Efficacy of 30% silver diamine fluoride compared to atraumatic restorative treatment on dentine caries arrestment in primary molars of preschool children: A 12-months parallel randomized controlled clinical trial. J Dent. 2019;88:103165. | Excluded; not orthodontics- related |
| 69 | Wang LF, Long H, Deng M, Xu H, Fang J, Fan Y, et al. Biofeedback treatment for sleep bruxism: a systematic review. Sleep Breath. 2014;18(2):235-42. | Excluded; not orthodontics- related |
| 70 | Yu M, Ma Y, Han F, Gao X. Long-term efficacy of mandibular advancement devices in the treatment of adult obstructive sleep apnea: A systematic review and meta- analysis. PLoS One. 2023;18(11):e0292832. | Excluded; not orthodontics- related |
| 71 | Casko JS, Vaden JL, Kokich VG, Damone J, James RD, Cangialosi TJ, et al. Objective grading system for dental casts and panoramic radiographs. American Board of Orthodontics. Am J Orthod Dentofacial Orthop. 1998;114(5):589-99. | Excluded; not clinical trial |
| 72 | El-Angbawi AM, Bearn DR, McIntyre GT. Comparing the effectiveness of the 0.018-inch versus the 0.022-inch bracket slot system in orthodontic treatment: study  protocol for a randomized controlled trial. Trials. 2014;15:389. | Excluded; not clinical trial |
| 73 | Huang GJ. Twin-block appliance is effective for the correction of Class II Division I malocclusion during mixed dentition. Journal of Evidence-Based Dental Practice. 2004;4(3):222-3. | Excluded; not clinical trial |
| 74 | Israel MH. An in vitro study comparing traditional and computer-aided bracket placement methods. 2007. | Excluded; not clinical trial |
| 75 | Seehra J, DiBiase AT, Patel S, Stephens R, Littlewood SJ, Spencer RJ, et al. Study protocol for the management of impacted maxillary central incisors: a multicentre randomised clinical trial: the iMAC Trial. Trials. 2022;23(1):787. | Excluded; not clinical trial |
| 76 | Thiruvenkatachari B, Hussain SA, Batra P, Vijayakumar C, Prathap CM. Reducing the burden of orthodontic care for children with clefts: evaluating the effectiveness  of pre-alveolar bone graft orthodontics in unilateral non-syndromic cleft patients (PABO study)- A study protocol for a multicentric randomised controlled trial. Trials. 2021;22(1):572. | Excluded; not clinical trial |
| 77 | Israel M, Kusnoto B, Evans CA, Begole E. A comparison of traditional and computer-aided bracket placement methods. Angle Orthod. 2011;81(5):828-35. | Excluded; not on humans |
| 78 | Alassiry AM. Orthodontic Retainers: A Contemporary Overview. J Contemp Dent Pract. 2019;20(7):857-62. | Excluded; no treatment |
| 79 | Anosike AN, Sanu OO, da Costa OO. Malocclusion and its impact on quality of life of school children in Nigeria. West Afr J Med. 2010;29(6):417-24. | Excluded; no treatment |
| 80 | Arruda AO. Occlusal indexes as judged by subjective opinions. Am J Orthod Dentofacial Orthop. 2008;134(5):671-5. | Excluded; no treatment |
| 81 | Baram D, Yang Y, Ren C, Wang Z, Wong RWK, Hägg U, et al. Orthodontic Treatment Need and the Psychosocial Impact of Malocclusion in 12-Year-Old Hong Kong Children. ScientificWorldJournal. 2019;2019:2685437. | Excluded; no treatment |
| 82 | Bootvong K, Liu Z, McGrath C, Haegg U, Wong RWK, Bendeus M, et al. Virtual model analysis as an alternative approach to plaster model analysis: reliability and  validity. European Journal of Orthodontics. 2010;32(5):589-95. | Excluded; no treatment |
| 83 | ElNaghy R, Hasanin M. Impact of malocclusions on oral health-related quality of life among adolescents. Evid Based Dent. 2023;24(3):140-1. | Excluded; no treatment |
| 84 | Järvinen S, Widström E. Determinants of costs of orthodontic treatment in the Finnish public health service. Swed Dent J. 2002;26(1):41-9. | Excluded; no treatment |
| 85 | Abbott S. A Retrospective Clinical Study to Compare The Efficacy of Bonded and Vacuum Formed Lower Arch Retainers. 2005. | Excluded; not randomized trial |
| 86 | Abdaljawwad AAM, Al-Groosh DH. Evaluation of treatment outcomes in orthodontic using the peer assessment rating (PAR) index: A clinical audit. International  Medical Journal. 2020;27(2):212-5. | Excluded; not randomized  trial |
| 87 | Abei Y, Nelson S, Amberman BD, Hans MG. Comparing orthodontic treatment outcome between orthodontists and general dentists with the ABO index. Am J Orthod Dentofacial Orthop. 2004;126(5):544-8. | Excluded; not randomized trial |
| 88 | Akinci Cansunar H, Uysal T. Comparison of orthodontic treatment outcomes in nonextraction, 2 maxillary premolar extraction, and 4 premolar extraction protocols with  the American Board of Orthodontics objective grading system. Am J Orthod Dentofacial Orthop. 2014;145(5):595-602. | Excluded; not randomized  trial |
| 89 | Akyalcin S, Rutkowski P, Arrigo M, Trotman CA, Kasper FK. Evaluation of current additive manufacturing systems for orthodontic 3-dimensional printing. Am J Orthod Dentofacial Orthop. 2021;160(4):594-602. | Excluded; not randomized trial |
| 90 | Alford TJ, Roberts WE, Hartsfield JK, Jr., Eckert GJ, Snyder RJ. Clinical outcomes for patients finished with the SureSmile™ method compared with conventional fixed orthodontic therapy. Angle Orthod. 2011;81(3):383-8. | Excluded; not randomized trial |
| 91 | Almeida AB, Leite IC, Melgaço CA, Marques LS. Dissatisfaction with dentofacial appearance and the normative need for orthodontic treatment: determinant factors. Dental Press J Orthod. 2014;19(3):120-6. | Excluded; not randomized trial |
| 92 | Anand M, Turpin DL, Jumani KS, Spiekerman CF, Huang GJ. Retrospective investigation of the effects and efficiency of self-ligating and conventional brackets. Am J Orthod Dentofacial Orthop. 2015;148(1):67-75. | Excluded; not randomized trial |
| 93 | Aragón MLC, Bichara LM, Flores-Mir C, Almeida G, Normando D. Efficiency of compensatory orthodontic treatment of mild Class III malocclusion with two different bracket systems. Dental Press J Orthod. 2017;22(6):49-55. | Excluded; not randomized trial |
| 94 | Bakos K. Upper incisors position changes after orthodontic treatment in Class I, II and III malocclusions. 2015. | Excluded; not randomized trial |
| 95 | Berndt J, Leone P, King G. Using teledentistry to provide interceptive orthodontic services to disadvantaged children. Am J Orthod Dentofacial Orthop 2008;134(5):700-6. | Excluded; not randomized trial |
| 96 | Berndt JN. Comparison of compliance and treatment outcome between Medicaid and non-Medicaid patients treated in a university setting. 2010. | Excluded; not randomized  trial |
| 97 | Berset GP, Eilertsen IM, Lagerström L, Espeland L, Stenvik A. Outcome of a scheme for specialist orthodontic care. Swed Dent J. 2000;24(1-2):39-48. | Excluded; not randomized trial |
| 98 | Berset GP. Outcome of a scheme for specialist orthodontic care. Swedish Dental Journal. 2000;24(1-2):39-48. | Excluded; not randomized  trial |
| 99 | Bjering R, Sandvik L, Midtbø M, Vandevska-Radunovic V. Stability of anterior tooth alignment 10 years out of retention. J Orofac Orthop. 2017;78(4):275-83. | Excluded; not randomized trial |
| 100 | Bjering R, Vandevska-Radunovic V. Occlusal changes during a 10-year posttreatment period and the effect of fixed retention on anterior tooth alignment. Am J Orthod Dentofacial Orthop 2018;154(4):487-94. | Excluded; not randomized trial |
| 101 | Blanck-Lubarsch M, Hohoff A, Wiechmann D, Stamm T. Orthodontic treatment of children/adolescents with special health care needs: an analysis of treatment length  and clinical outcome. BMC Oral Health. 2014;14:67. | Excluded; not randomized  trial |
| 102 | Bock N, Ruehl J, Ruf S. Orthodontic Class II:1 treatment-efficiency and outcome quality of Herbst-multibracket appliance therapy. Clinical Oral Investigations. 2018;22(5):2005-11. | Excluded; not randomized trial |
| 103 | Calheiros AdA, Miguel JAM, Moura PM, Almeida MAdO. Tratamento da má oclusão de Classe II de Angle em duas fases: avaliação da efetividade e eficácia por meio do índice PAR. Rev dent press ortodon ortopedi facial. 2008;13(1):43-53. | Excluded; not randomized trial |
| 104 | Cansunar HA, Uysal T. Comparison of orthodontic treatment outcomes in nonextraction, 2 maxillary premolar extraction, and 4 premolar extraction protocols with the American Board of Orthodontics objective grading system. Am J Orthod Dentofacial Orthop 2014;145(5):595-602. | Excluded; not randomized trial |
| 105 | Cansunar HA, Uysal T. Outcomes of different Class II treatments Comparisons using the American Board of Orthodontics Model Grading System. Journal of Orofacial Orthopedics-Fortschritte Der Kieferorthopadie. 2016;77(4):233-41. | Excluded; not randomized trial |
| 106 | Cardoso PC, Mecenas P, Normando D. The impact of the loss of first permanent molars on the duration of treatment in patients treated with orthodontic space closure and without skeletal anchorage. Prog Orthod. 2022;23(1):32. | Excluded; not randomized trial |
| 107 | Carvajal-Flórez A, Barbosa-Lis DM, Zapata-Noreña OA, Marín-Velásquez JA, Afanador-Bayona SA. Orthodontic treatment outcomes obtained by application of a  finishing protocol. Dental Press J Orthod. 2016;21(2):88-94. | Excluded; not randomized  trial |
| 108 | Chalabi O, Preston CB, Al-Jewair TS, Tabbaa S. A comparison of orthodontic treatment outcomes using the Objective Grading System (OGS) and the Peer Assessment Rating (PAR) index. Aust Orthod J. 2015;31(2):157-64. | Excluded; not randomized trial |
| 109 | Chao-Feng LIU, Yan-Hua XU, Yan LIU. Curative effect evaluation by using Andrews' six elements for completed orthodontic treatment cases. West China Journal of  Stomatology. 2021(6):48-52. | Excluded; not randomized  trial |
| 110 | Cifter M, Celikel ADG, Cekici A. Effects of vacuum-formed retainers on periodontal status and their retention efficiency. Am J Orthod Dentofacial Orthop 2017;152(6):830-5. | Excluded; not randomized trial |
| 111 | Coenen FA, Bartz JR, Niederau C, Craveiro RB, Knaup I, Wolf M. Orthodontic treatment quality evaluated by partially automated digital IOTN and PAR index  determination: a retrospective multicentre study. European Journal of Orthodontics. 2023;45(3):308-16. | Excluded; not randomized  trial |
| 112 | Cook DR, Harris EF, Vaden JL. Comparison of university and private-practice orthodontic treatment outcomes with the American Board of Orthodontics objective grading system. Am J Orthod Dentofacial Orthop. 2005;127(6):707-12. | Excluded; not randomized trial |
| 113 | Dayan D. Accuracy of 3-dimensional Printing of Orthodontic Study Models by the Objet30 OrthoDesk Printer. 2015. | Excluded; not randomized  trial |

| 114 | de Albuquerque Calheiros A, Mendes Miguel JA, Moura PM, de Oliveira Almeida MA. Two phase treatment of Angle Class II: Effectiveness and efficacy evaluation using the PAR index. Revista Dental Press de Ortodontia e Ortopedia Facial. 2008;13(1):43-53. | Excluded; not randomized trial |
| --- | --- | --- |
| 115 | Deguchi T, Kurosaka H, Oikawa H, Kuroda S, Takahashi I, Yamashiro T, et al. Comparison of orthodontic treatment outcomes in adults with skeletal open bite  between conventional edgewise treatment and implant-anchored orthodontics. Am J Orthod Dentofacial Orthop. 2011;139(4 Suppl):S60-8. | Excluded; not randomized  trial |
| 116 | Dietz DA. An analysis of orthodontic treatment outcomes in Alberta using the PAR Index and the American Board of Orthodontics Objective Grading System. 2004. | Excluded; not randomized trial |
| 117 | Dolce C, Mansour DA, McGorray SP, Wheeler TT. Intrarater agreement about the etiology of Class II malocclusion and treatment approach. Am J Orthod Dentofacial  Orthop. 2012;141(1):17-23. | Excluded; not randomized  trial |
| 118 | Fadiga MS, Diouf JS, Diop Ba K, Gueye I, Ngom PI, Diagne F. The PAR index for evaluation of treatment outcomes in orthodontics: a clinical audit of 50 cases. Int Orthod. 2014 Mar;12(1):84-99. | Excluded; not randomized trial |
| 119 | Fleming JW, Buschang PH, Kim KB, Oliver DR. Posttreatment occlusal variability among Angle Class I nonextraction patients. Angle Orthodontist. 2008;78(4):625-30. | Excluded; not randomized  trial |
| 120 | Freitas KM, Janson G, Tompson B, de Freitas MR, Simão TM, Valarelli FP, et al. Posttreatment and physiologic occlusal changes comparison. Angle Orthod. 2013;83(2):239-45. | Excluded; not randomized trial |
| 121 | Frick CJ, Deng HH, English JD, Jacob HB, Kuang T, Grissom MK, et al. Clinical feasibility evaluation of digital dental articulation for three-piece maxillary orthognathic  surgery: a proof-of-concept study. Int J Oral Maxillofac Surg. 2022;51(8):1043-9. | Excluded; not randomized  trial |
| 122 | Gonzalez-Gil-de-Bernabe P, Bellot-Arcís C, Montiel-Company JM, Gandía-Franco JL. Evaluation of treatment outcomes in a 3 years post-graduate orthodontic program using the peer assessment rating (par). Journal of Clinical and Experimental Dentistry. 2014;6(4):e364-e8. | Excluded; not randomized trial |
| 123 | Graf I, Bock NC, Bartzela T, Röper V, Schumann U, Reck K, et al. Quality of orthodontic care—A multicenter cohort study in Germany: Part 1: Evaluation of  effectiveness of orthodontic treatments and predictive factors. Journal of Orofacial Orthopedics. 2022;83(5):291-306. | Excluded; not randomized  trial |
| 124 | Graf I, Puppe C, Schwarze J, Höfer K, Christ H, Braumann B. Evaluation of effectiveness and stability of aligner treatments using the Peer Assessment Rating Index. J Orofac Orthop. 2021;82(1):23-31. | Excluded; not randomized trial |
| 125 | Hajeer MY. Assessment of dental arches in patients with Class II division 1 and division 2 malocclusions using 3D digital models in a Syrian sample. Eur J Paediatr  Dent. 2014;15(2):151-7. | Excluded; not randomized  trial |
| 126 | Heinz J, Stewart K, Ghoneima A. Evaluation of two-dimensional lateral cephalogram and three-dimensional cone beam computed tomography superimpositions: a comparative study. Int J Oral Maxillofac Surg. 2019;48(4):519-25. | Excluded; not randomized trial |
| 127 | Horriat M, Bailey N, Atout B, Santos PB, Pinheiro F. American Board of Orthodontics (ABO) Discrepancy Index and peer assessment rating (PAR) index with models  versus photographs. J World Fed Orthod. 2022;11(3):83-9. | Excluded; not randomized  trial |
| 128 | Hsieh TJ, Pinskaya Y, Roberts WE. Assessment of orthodontic treatment outcomes: Early treatment versus late treatment. Angle Orthodontist. 2005;75(2):162-70. | Excluded; not randomized trial |
| 129 | Janson G, Barros SEC, de Freitas MR, Henriques JFC, Pinzan A. Class II treatment efficiency in maxillary premolar extraction and nonextraction protocols. Am J  Orthod Dentofacial Orthop 2007;132(4):490-8. | Excluded; not randomized  trial |
| 130 | Kaygisiz E, Uzuner FD, Taner L. A Comparison of Three Orthodontic Treatment Indices with Regard to Angle Classification. J Clin Pediatr Dent. 2016;40(2):169-74. | Excluded; not randomized trial |
| 131 | Kerosuo H, Heikinheimo K, Nystrom M, Vakiparta M. Outcome and long-term stability of an early orthodontic treatment strategy in public health care. European  Journal of Orthodontics. 2013;35(2):183-9. | Excluded; not randomized  trial |
| 132 | Kerosuo H, Vakiparta M, Nystrom M, Heikinheimo K. The seven-year outcome of an early orthodontic treatment strategy. Journal of Dental Research. 2008;87(6):584- 8. | Excluded; not randomized trial |
| 133 | Khandakji MN, Ghafari JG. Evaluation of commonly used occlusal indices in determining orthodontic treatment need. Eur J Orthod. 2020;42(1):107-14. | Excluded; not randomized  trial |
| 134 | Kim E, Sherf N, Lamichane M, Levine S, Allareddy V, Ellenikiotis Y, et al. A comparison of SureSmile™, Insignia™, and Invisalign™, in treating non-extraction cases of mild to moderate crowding: a prospective clinical trial. Australasian Orthodontic Journal. 2022;38(2):290-306. | Excluded; not randomized trial |
| 135 | Kim JC, Mascarenhas AK, Joo BH, Vig KWL, Beck FM, Vig PS. Cephalometric variables as predictors of Class II treatment outcome. Am J Orthod Dentofacial Orthop 2000;118(6):636-40. | Excluded; not randomized trial |
| 136 | King GJ, Kiyak HA, Greenlee GM, Huang GJ, Spiekerman CF. Medicaid and privately financed orthodontic patients have similar occlusal and psychosocial outcomes. J Public Health Dent. 2012;72(2):94-103. | Excluded; not randomized trial |
| 137 | Lagerström L, Fornell AC, Stenvik A. Outcome of a scheme for specialist orthodontic care, a follow-up study in 31-year-olds. Swed Dent J. 2011;35(1):41-7. | Excluded; not randomized trial |
| 138 | Li Y, Deng S, Mei L, Li Z, Zhang X, Yang C, et al. Prevalence and severity of apical root resorption during orthodontic treatment with clear aligners and fixed appliances: a cone beam computed tomography study. Progress in Orthodontics. 2020;21(1 C7 - 1). | Excluded; not randomized trial |
| 139 | Lieber WS, Carlson SK, Baumrind S, Poulton DR. Clinical use of the ABO-Scoring Index: reliability and subtraction frequency. Angle Orthod. 2003;73(5):556-64. | Excluded; not randomized trial |
| 140 | Liu C-F, Xu Y-H, Liu Y. Curative effect evaluation by using Andrews' six elements for completed orthodontic treatment cases. Hua xi kou qiang yi xue za zhi = Huaxi kouqiang yixue zazhi = West China journal of stomatology. 2021;39(1):48-52. | Excluded; not randomized trial |
| 141 | Liu S, Oh H, Chambers DW, Xu T, Baumrind S. Interpreting weightings of the peer assessment rating index and the discrepancy index across contexts on Chinese patients. Eur J Orthod. 2018;40(2):157-63. | Excluded; not randomized trial |
| 142 | Liu SQ, Shen G, Bai D, Zhou H, Li S, Chen WJ, et al. [Consistency of the subjective evaluation of malocclusion severity by the Chinese orthodontic experts]. Beijing Da Xue Xue Bao Yi Xue Ban. 2012;44(1):98-102. | Excluded; not randomized trial |
| 143 | Machibya FM, Bao X, Zhao L, Hu M. Treatment time, outcome, and anchorage loss comparisons of self-ligating and conventional brackets. Angle Orthodontist. 2013;83(2):280-5. | Excluded; not randomized trial |
| 144 | Malik OH, Stevenson H, Mandall NA, Alsaeed MA. Effectiveness of photographs of study models for IOTN scoring. Australian Orthodontic Journal. 2016;32(2):221-8. | Excluded; not randomized trial |
| 145 | Marques LS, Freitas Junior N, Pereira LJ, Ramos-Jorge ML. Quality of orthodontic treatment performed by orthodontists and general dentists. Angle Orthod. 2012;82(1):102-6. | Excluded; not randomized trial |
| 146 | McGuinness NJ, McDonald JP. The influence of operator changes on orthodontic treatment times and results in a postgraduate teaching environment. Eur J Orthod. 1998;20(2):159-67. | Excluded; not randomized trial |
| 147 | McMullan RE. An audit of 'early debond' cases in the national outcomes audit of patients treated with upper and lower fixed appliances by Consultant Orthodontists in theUK. Journal of Orthodontics. 2005;32(4):257-61. | Excluded; not randomized trial |
| 148 | Mejía-Rodríguez AM, Álvarez-Varela E, Roldan SI. Evaluación de la confiabilidad del índice PAR en modelos de estudio físicos y digitales. CES odontol. 2013;26(2):49-58. | Excluded; not randomized trial |
| 149 | Mendes LM. Influência em longo prazo dos protocolos de tratamento da Classe II na atratividade do perfil. 2012. p. 179-. | Excluded; not randomized trial |
| 150 | Mirabelli JT, Huang GJ, Siu CH, King GJ, Omnell L. The effectiveness of phase I orthodontic treatment in a Medicaid population. Am J Orthod Dentofacial Orthop 2005;127(5):592-8. | Excluded; not randomized trial |
| 151 | Miranda F. Qualitative longitudinal assessment of normal occlusion maturation: 40 years of follow-up. 2016. p. 78-. | Excluded; not randomized  trial |
| 152 | Mommaerts MY, Michiels ML, De Pauw GA. A 2-year outcome audit of a versatile orthodontic bone anchor. J Orthod. 2005;32(3):175-81. | Excluded; not randomized trial |
| 153 | Moreira FC, Vaz LG, Guastaldi AC, English JD, Jacob HB. Potentialities and limitations of computer-aided design and manufacturing technology in the nonextraction treatment of Class I malocclusion. Am J Orthod Dentofacial Orthop. 2021;159(1):86-96. | Excluded; not randomized trial |
| 154 | Mostafa RY, Bous RM, Hans MG, Valiathan M, Copeland GE, Palomo JM. Effects of Case Western Reserve University's transverse analysis on the quality of  orthodontic treatment. Am J Orthod Dentofacial Orthop 2017;152(2):178-92. | Excluded; not randomized  trial |
| 155 | Murphy SJ, Lee S, Scharm JC, Kim S, Amin AA, Wu TH, et al. Comparison of maxillary anterior tooth movement between Invisalign and fixed appliances. Am J Orthod Dentofacial Orthop 2023;164(1):24-33. | Excluded; not randomized trial |
| 156 | Nagshabandi R. Accuracy of Digital Models Using the American Board of Orthodontics Cast-radiographic Evaluation. 2018. | Excluded; not randomized  trial |
| 157 | Nett BC, Huang GJ. Long-term posttreatment changes measured by the American Board of Orthodontics objective grading system. Am J Orthod Dentofacial Orthop. 2005;127(4):444-50; quiz 516. | Excluded; not randomized trial |
| 158 | O'Brien K, Wright J, Conboy F, Appelbe P, Bearn D, Caldwell S, et al. Prospective, multi-center study of the effectiveness of orthodontic/orthognathic surgery care in the United Kingdom. Am J Orthod Dentofacial Orthop 2009;135(6):709-14. | Excluded; not randomized trial |
| 159 | O'Brien K. An Analysis of the Effectiveness of the Provision of Orthodontic Treatment by the Hospital Orthodontic Service of England and Wales. 1991. | Excluded; not randomized trial |
| 160 | Onyeaso CO, Begole EA. Orthodontic treatment--improvement and standards using the peer assessment rating index. Angle Orthod. 2006;76(2):260-4. | Excluded; not randomized trial |
| 161 | Onyeaso CO, Begole EA. Relationship between index of complexity, outcome and need, dental aesthetic index, peer assessment rating index, and American Board of Orthodontics objective grading system. Am J Orthod Dentofacial Orthop. 2007;131(2):248-52. | Excluded; not randomized trial |
| 162 | Pangrazio-Kulbersh V, Kaczynski R, Shunock M. Early treatment outcome assessed by the Peer Assessment Rating index. Am J Orthod Dentofacial Orthop. 1999;115(5):544-50. | Excluded; not randomized trial |
| 163 | Pariskou A, Athanasiou AE. Assessing treatment outcomes of a graduate orthodontic program. Open Dentistry Journal. 2018;12(1):896-903. | Excluded; not randomized  trial |
| 164 | Park H, Boley JC, Alexander RA, Buschang PH. Age-related long-term posttreatment occlusal and arch changes. Angle Orthod. 2010;80(2):247-53. | Excluded; not randomized trial |
| 165 | Park Y, Hartsfield JK, Katona TR, Eugene Roberts W. Tooth positioner effects on occlusal contacts and treatment outcomes. Angle Orthod. 2008;78(6):1050-6. | Excluded; not randomized  trial |

| 166 | Peppers EC, Leggitt VL, Caruso JM, Neufeld R, Green J. The effect of changes in primary attending doctor coverage frequency on orthodontic treatment time and results. Angle Orthodontist. 2015;85(6):1051-6. | Excluded; not randomized trial |
| --- | --- | --- |
| 167 | Plaza SP, Aponte CM, Bejarano SR, Martínez YJ, Serna S, Barbosa-Liz DM. Relationship between the Dental Aesthetic Index and Discrepancy Index. J Orthod.  2020;47(3):213-22. | Excluded; not randomized  trial |
| 168 | Poulton D, Vlaskalic V, Baumrind S. Treatment outcomes in 4 modes of orthodontic practice. Am J Orthod Dentofacial Orthop. 2005;127(3):351-4. | Excluded; not randomized trial |
| 169 | Rasheed B, Khalid A, Wahab A, Zahid A, Ahmed A, Sarfraz T. EXAMINATION OF THE EFFECT OF PERSISTENT MOLAR LOSS ON LENGTH OF ORTHODONTIC  THERAPY FOR SPACE CLOSURE. NeuroQuantology. 2023;21(5):1438-46. | Excluded; not randomized  trial |
| 170 | Read MJ, Deacon S, O'Brien K. A prospective cohort study of a clip-on fixed functional appliance. Am J Orthod Dentofacial Orthop. 2004;125(4):444-9. | Excluded; not randomized trial |
| 171 | Richmond S, Ikonomou C, Williams B, Ramel S, Rolfe B, Kurol J. Orthodontic treatment standards in a public group practice in Sweden. Swedish Dental Journal.  2001;25(4):137-44. | Excluded; not randomized  trial |
| 172 | Richmond S. A Critical Evaluation of Orthodontic Treatment in the General Dental Services of England and Wales. 1990. | Excluded; not randomized trial |
| 173 | Rodrigues-Garcia RC, Sakai S, Rugh JD, Hatch JP, Tiner BD, van Sickels JE, et al. Effects of major Class II occlusal corrections on temporomandibular signs and  symptoms. J Orofac Pain. 1998;12(3):185-92. | Excluded; not randomized  trial |
| 174 | Rooney C, Dhaliwal H, Hodge T. Orthodontic therapists-has their introduction affected outcomes? British Dental Journal. 2016;221(7):421-4. | Excluded; not randomized trial |
| 175 | Saxe AK, Louie LJ, Mah J. Efficiency and effectiveness of SureSmile. World J Orthod. 2010;11(1):16-22. | Excluded; not randomized  trial |
| 176 | Scott SA, Freer TJ. Visual application of the American Board of Orthodontics Grading System. Aust Orthod J. 2005;21(1):55-60. | Excluded; not randomized trial |
| 177 | Sepanian VF, Paulsson-Bjornsson L, Kjellberg H. A long-term controlled follow-up study of objective treatment need on young adults treated with functional  appliances. Swedish Dental Journal. 2014;38(1):39-46. | Excluded; not randomized  trial |
| 178 | Sfondrini MF, Zampetti P, Luscher G, Gandini P, Gandía-Franco JL, Scribante A. Orthodontic Treatment and Healthcare Goals: Evaluation of Multibrackets Treatment Results Using PAR Index (Peer Assessment Rating). Healthcare. 2021;8(4):1-11. | Excluded; not randomized trial |
| 179 | Sharma V, Sengupta J. Modifications to increase efficiency of the Begg orthodontic technique. Medical Journal Armed Forces India. 2009;65(2):118-22. | Excluded; not randomized  trial |
| 180 | Shim J, Ho KCJ, Shim BC, Metaxas A, Somogyi-Ganss E, Di Sipio R, et al. Impact of post-orthodontic dental occlusion on masticatory performance and chewing efficiency. European Journal of Orthodontics. 2020;42(6):587-95. | Excluded; not randomized trial |
| 181 | Sjogren APG, Lindgren JE, Huggare JAV. Orthodontic Study Cast Analysis-Reproducibility of Recordings and Agreement Between Conventional and 3D Virtual  Measurements. Journal of Digital Imaging. 2010;23(4):482-92. | Excluded; not randomized  trial |
| 182 | Song G, Wang X, Teng F, Yu M, Pei R, Xu T. Consistency of peer assessment rating index by computer 3D digital measurement and manual measurement. Chinese Journal of Orthodontics. 2019;26(1):22-6. | Excluded; not randomized trial |
| 183 | Song GY, Baumrind S, Zhao ZH, Ding Y, Bai YX, Wang L, et al. Validation of the American Board of Orthodontics Objective Grading System for assessing the  treatment outcomes of Chinese patients. Am J Orthod Dentofacial Orthop. 2013;144(3):391-7. | Excluded; not randomized  trial |
| 184 | Song GY, Jiang RP, Zhang XY, Liu SQ, Yu XN, Chen Q, et al. [Validation of subjective and objective evaluation methods for orthodontic treatment outcome]. Beijing Da Xue Xue Bao Yi Xue Ban. 2015;47(1):90-7. | Excluded; not randomized trial |
| 185 | Song G-Y, Zhao Z-H, Ding Y, Bai Y-X, Wang L, He H, et al. Reliability assessment and correlation analysis of evaluating orthodontic treatment outcome in Chinese  patients. International Journal of Oral Science. 2014;6(1):50-5. | Excluded; not randomized  trial |
| 186 | Struble BH, Huang GJ. Comparison of prospectively and retrospectively selected American Board of Orthodontics cases. Am J Orthod Dentofacial Orthop. 2010;137(1):6.e1-8; discussion 6-8. | Excluded; not randomized trial |
| 187 | Teh LH, Kerr WJ, McColl JH. Orthodontic treatment with fixed appliances in the General Dental Service in Scotland. J Orthod. 2000;27(2):175-80. | Excluded; not randomized trial |
| 188 | Teh L-H. Orthodontic Treatment With Fixed Appliances in The General Dental Service of Scotland. 1998. | Excluded; not randomized trial |
| 189 | Thiruvenkatachari B, Javidi H, Griffiths SE, Shah AA, Sandler J. Extraction of maxillary canines: Esthetic perceptions of patient smiles among dental professionals and laypeople. Am J Orthod Dentofacial Orthop. 2017;152(4):509-15. | Excluded; not randomized trial |
| 190 | Turbill EA, Richmond S, Wright JL. A critical assessment of high-earning orthodontists in the General Dental Services of England and Wales (1990-1991). Br J Orthod. 1998;25(1):47-54. | Excluded; not randomized trial |
| 191 | Turbill EA. National orthodontic standards in the General Dental Services: An assessment of factors affecting outcome and the effectiveness of the current methods of monitoring. 1996. | Excluded; not randomized trial |
| 192 | Vaiciunaite R, Mitalauskiene A, Vasiliauskas A. The relationship between congenital cleft lip and palate malformation, skeletal and dental occlusal anomalies, and the influence of its treatment on affected patients' oral health-related quality of life (OHRQoL). Stomatologija. 2020;22(4):116-9. | Excluded; not randomized trial |
| 193 | Valladares Neto J. Avaliação da qualidade de vida em adultos com deformidade dentofacial de Classe III. 2013. p. 100-. | Excluded; not randomized trial |
| 194 | Vasilakou N, Araujo EA, Kim KB, Oliver DR. Quantitative assessment of the effectiveness of phase 1 orthodontic treatment using the American Board of Orthodontics Discrepancy Index. Am J Orthod Dentofacial Orthop. 2016;150(6):997-1004. | Excluded; not randomized trial |
| 195 | Vu J, Pancherz H, Schwestka-Polly R, Wiechmann D. Correction of Class II, Division 2 malocclusions using a completely customized lingual appliance and the Herbst device. J Orofac Orthop. 2012;73(3):225-35. | Excluded; not randomized trial |
| 196 | Wes Fleming J, Buschang PH, Kim KB, Oliver DR. Posttreatment occlusal variability among angle Class I nonextraction patients. Angle Orthod. 2008;78(4):625-30. | Excluded; not randomized trial |
| 197 | Xu K, Hasan SE, Chen G, Liu S, Chambers DW, Xu T. Measuring orthodontic treatment impact: Description or judgment, challenge or result. Am J Orthod Dentofacial Orthop. 2021;159(5):e389-e97. | Excluded; not randomized trial |
| 198 | Yadav VK. Efficacy of Clear Aligners in Orthodontic Tooth Movement – An in Vivo Prospective Study. 2018. | Excluded; not randomized trial |
| 199 | Zimmer B, Gaida S, Dathe H. Compensation of skeletal Class III malocclusion by isolated extraction of mandibular teeth: Part 2: Skeletal, dentoalveolar and soft tissue parameters in comparison with nonextraction Class III therapies. J Orofac Orthop. 2016;77(2):119-28. | Excluded; not randomized trial |
| 200 | Zimmer B, Schenk-Kazan S. Dental compensation for skeletal Class III malocclusion by isolated extraction of mandibular teeth. Part 1: Occlusal situation 12 years after completion of active treatment. J Orofac Orthop. 2015;76(3):251-64. | Excluded; not randomized trial |
| 201 | Al Rahma WJ, Kaklamanos EG, Athanasiou AE. Performance of Hawley-type retainers: a systematic review of randomized clinical trials. Eur J Orthod. 2018;40(2):115-25. | Excluded; review |
| 202 | Alwafi A, Bichu YM, Avanessian A, Zou B. Overview of systematic reviews and meta-analyses assessing the predictability and clinical effectiveness of clear aligner therapy. Dentistry Review. 2023;3(4). | Excluded; review |
| 203 | Bakdach WMM, Hadad R. Linear and angular transfer accuracy of labial brackets using three dimensional-printed indirect bonding trays: A systematic review and  meta-analysis. Int Orthod. 2022;20(1):100612. | Excluded; review |
| 204 | Barone S, Antonelli A, Bocchino T, Cevidanes L, Michelotti A, Giudice A. Managing Mandibular Second Molar Impaction: A Systematic Review and Meta-Analysis. J Oral Maxillofac Surg. 2023;81(11):1403-21. | Excluded; review |
| 205 | Chen SS, Greenlee GM, Kim JE, Smith CL, Huang GJ. Systematic review of self-ligating brackets. Am J Orthod Dentofacial Orthop. 2010;137(6):726.e1-.e18; discussion -7. | Excluded; review |
| 206 | Chok A, Dallel I, Ommezine M, Tobji S, Ben Amor A. [Quality of finishing occlusion and long-term stability of orthodontic treatments with premolar extractions: A  systematic review]. L' Orthodontie francaise. 2020;91(3):191-5. | Excluded; review |
| 207 | Elias KG, Sivamurthy G, Bearn DR. Extraction vs nonextraction orthodontic treatment: a systematic review and meta-analysis. Angle Orthod. 2024;94(1):83-106. | Excluded; review |
| 208 | Gandhi R, Jnaneshwar P, Venkatesan K, Devasahayam D, Rajaram K, Azharudeen RM, et al. Assessment of the outcomes and stability after mandibular incisor extraction in orthodontic patients: A systematic review and meta-analysis. Journal of Dental Research, Dental Clinics, Dental Prospects. 2023;17(2):71-80. | Excluded; review |
| 209 | Gudelevičiūtė I, Spaičytė N, Smailienė D. Skeletal and dental maxillary morphological characteristics in patients with impacted canines: systematic review and meta-  analysis. Eur J Orthod. 2023;45(6):832-41. | Excluded; review |
| 210 | Hamedi Sangsari A, Sadr-Eshkevari P, Al-Dam A, Friedrich RE, Freymiller E, Rashad A. Surgically Assisted Rapid Palatomaxillary Expansion With or Without Pterygomaxillary Disjunction: A Systematic Review and Meta-Analysis. J Oral Maxillofac Surg. 2016;74(2):338-48. | Excluded; review |
| 211 | Harrison JE, O'Brien KD, Worthington HV. Orthodontic treatment for prominent upper front teeth in children. Cochrane Database Syst Rev. 2007(3):Cd003452. | Excluded; review |
| 212 | Kaklamanos EG, Kourakou M, Kloukos D, Doulis I, Kavvadia S. Performance of clear vacuum-formed thermoplastic retainers depending on retention protocol: a systematic review. Odontology. 2017;105(2):237-47. | Excluded; review |
| 213 | Ke Y, Zhu Y, Zhu M. A comparison of treatment effectiveness between clear aligner and fixed appliance therapies. BMC Oral Health. 2019;19(1):24. | Excluded; review |
| 214 | Kloukos D, Fudalej P, Sequeira-Byron P, Katsaros C. Maxillary distraction osteogenesis versus orthognathic surgery for cleft lip and palate patients. Cochrane Database Syst Rev. 2018;8(8):Cd010403. | Excluded; review |
| 215 | Mai W, He J, Meng H, Jiang Y, Huang C, Li M, et al. Comparison of vacuum-formed and Hawley retainers: a systematic review. Am J Orthod Dentofacial Orthop.  2014;145(6):720-7. | Excluded; review |
| 216 | Maizeray R, Wagner D, Lefebvre F, Lévy-Bénichou H, Bolender Y. Is there any difference between conventional, passive and active self-ligating brackets? A systematic review and network meta-analysis. Int Orthod. 2021;19(4):523-38. | Excluded; review |
| 217 | Millett DT, Cunningham SJ, O'Brien KD, Benson PE, de Oliveira CM. Treatment and stability of class II division 2 malocclusion in children and adolescents: a  systematic review. Am J Orthod Dentofacial Orthop. 2012;142(2):159-69.e9. | Excluded; review |
| 218 | Mousoulea S, Papageorgiou SN, Eliades T. Treatment effects of various prescriptions and techniques for fixed orthodontic appliances : A systematic review. J Orofac Orthop. 2017;78(5):403-14. | Excluded; review |

| 219 | Outhaisavanh S, Liu Y, Song J. The origin and evolution of the Hawley retainer for the effectiveness to maintain tooth position after fixed orthodontic treatment compare to vacuum-formed retainer: A systematic review of RCTs. Int Orthod. 2020;18(2):225-36. | Excluded; review |
| --- | --- | --- |
| 220 | Papageorgiou SN, Höchli D, Eliades T. Outcomes of comprehensive fixed appliance orthodontic treatment: A systematic review with meta-analysis and methodological  overview. Korean Journal of Orthodontics. 2017;47(6):401-13. | Excluded; review |
| 221 | Papageorgiou SN, Koletsi D, Iliadi A, Peltomaki T, Eliades T. Treatment outcome with orthodontic aligners and fixed appliances: a systematic review with meta- analyses. Eur J Orthod. 2020;42(3):331-43. | Excluded; review |
| 222 | Papageorgiou SN, Kutschera E, Memmert S, Gölz L, Jäger A, Bourauel C, et al. Effectiveness of early orthopaedic treatment with headgear: a systematic review and  meta-analysis. Eur J Orthod. 2017;39(2):176-87. | Excluded; review |
| 223 | Shoukat Ali U, Zafar K, Hoshang Sukhia R, Fida M, Ahmed A. Effect of bonded and removable retainers on occlusal settling after orthodontic treatment: A systematic review and meta-analysis. Dent Med Probl. 2023;60(2):327-34. | Excluded; review |
| 224 | Sunnak R, Johal A, Fleming PS. Is orthodontics prior to 11 years of age evidence-based? A systematic review and meta-analysis. J Dent. 2015;43(5):477-86. | Excluded; review |
| 225 | Thiruvenkatachari B, Harrison JE, Worthington HV, O'Brien KD. Orthodontic treatment for prominent upper front teeth (Class II malocclusion) in children. Cochrane Database Syst Rev. 2013(11):Cd003452. | Excluded; review |
| 226 | Tsichlaki A, Chin SY, Pandis N, Fleming PS. How long does treatment with fixed orthodontic appliances last? A systematic review. Am J Orthod Dentofacial Orthop. 2016;149(3):308-18. | Excluded; review |
| 227 | Woon SC, Thiruvenkatachari B. Early orthodontic treatment for Class III malocclusion: A systematic review and meta-analysis. Am J Orthod Dentofacial Orthop.  2017;151(1):28-52. | Excluded; review |
| 228 | Zheng M, Liu R, Ni Z, Yu Z. Efficiency, effectiveness and treatment stability of clear aligners: A systematic review and meta-analysis. Orthod Craniofac Res. 2017;20(3):127-33. | Excluded; review |
| 229 | Campbell C, Millett D, Kelly N, Cooke M, Cronin M. Frankel 2 appliance versus the Modified Twin Block appliance for Phase 1 treatment of Class II division 1  malocclusion in children and adolescents: A randomized clinical trial. Angle Orthod. 2020;90(2):202-8. | Excluded; not (only) fixed  appliance treatment |
| 230 | Clements KM, Bollen AM, Huang G, King G, Hujoel P, Ma T. Activation time and material stiffness of sequential removable orthodontic appliances. Part 2: Dental improvements. Am J Orthod Dentofacial Orthop. 2003;124(5):502-8. | Excluded; not (only) fixed appliance treatment |
| 231 | Jolley CJ, Huang GJ, Greenlee GM, Spiekerman C, Kiyak HA, King GJ. Dental effects of interceptive orthodontic treatment in a Medicaid population: interim results  from a randomized clinical trial. Am J Orthod Dentofacial Orthop. 2010;137(3):324-33. | Excluded; not (only) fixed  appliance treatment |
| 232 | King GJ, McGorray SP, Wheeler TT, Dolce C, Taylor M. Comparison of peer assessment ratings (PAR) from 1-phase and 2-phase treatment protocols for Class II malocclusions. Am J Orthod Dentofacial Orthop. 2003;123(5):489-96. | Excluded; not (only) fixed appliance treatment |
| 233 | King GJ, Spiekerman CF, Greenlee GM, Huang GJ. Randomized clinical trial of interceptive and comprehensive orthodontics. J Dent Res. 2012;91(7 Suppl):59s-64s. | Excluded; not (only) fixed  appliance treatment |
| 234 | Krusinskiene V, Kiuttu P, Julku J, Silvola AS, Kantomaa T, Pirttiniemi P. A randomized controlled study of early headgear treatment on occlusal stability--a 13 year follow-up. Eur J Orthod. 2008;30(4):418-24. | Excluded; not (only) fixed appliance treatment |
| 235 | Mandall N, Cousley R, DiBiase A, Dyer F, Littlewood S, Mattick R, et al. Early class III protraction facemask treatment reduces the need for orthognathic surgery: a  multi-centre, two-arm parallel randomized, controlled trial. J Orthod. 2016;43(3):164-75. | Excluded; not (only) fixed  appliance treatment |
| 236 | Mandall N, DiBiase A, Littlewood S, Nute S, Stivaros N, McDowall R, et al. Is early Class III protraction facemask treatment effective? A multicentre, randomized, controlled trial: 15-month follow-up. J Orthod. 2010;37(3):149-61. | Excluded; not (only) fixed appliance treatment |
| 237 | Mandall NA, Cousley R, DiBiase A, Dyer F, Littlewood S, Mattick R, et al. Is early class III protraction facemask treatment effective?Amulticentre, randomized,  controlled trial: 3-Year follow-up. Journal of Orthodontics. 2012;39(3):176-85. | Excluded; not (only) fixed  appliance treatment |
| 238 | Mehyar L, Sandler J, Thiruvenkatachari B. Does observational study on the effectiveness of the Twin Blocks overestimate or underestimate the results? A comparative analysis of retrospective samples versus randomized controlled trial. J World Fed Orthod. 2021;10(2):43-8. | Excluded; not (only) fixed appliance treatment |
| 239 | O'Brien K, Wright J, Conboy F, et al. Early treatment for Class II Division 1 malocclusion with the Twin-block appliance: a multi-center, randomized, controlled trial. Am  J Orthod Dentofacial Orthop. 2009 May;135(5):573-9. | Excluded; not (only) fixed  appliance treatment |
| 240 | Pavlow SS, McGorray SP, Taylor MG, Dolce C, King GJ, Wheeler TT. Effect of early treatment on stability of occlusion in patients with Class II malocclusion. Am J Orthod Dentofacial Orthop. 2008;133(2):235-44. | Excluded; not (only) fixed appliance treatment |
| 241 | Pirttiniemi P, Kantomaa T, Mäntysaari R, Pykäläinen A, Krusinskiene V, Laitala T, et al. The effects of early headgear treatment on dental arches and craniofacial morphology: an 8 year report of a randomized study. Eur J Orthod. 2005;27(5):429-36. | Excluded; not (only) fixed appliance treatment |
| 242 | Thickett E, Power S. A randomized clinical trial of thermoplastic retainer wear. Eur J Orthod. 2010;32(1):1-5. | Excluded; not (only) fixed appliance treatment |
| 243 | Tulloch JF, Proffit WR, Phillips C. Outcomes in a 2-phase randomized clinical trial of early Class II treatment. Am J Orthod Dentofacial Orthop. 2004 Jun;125(6):657- 67. | Excluded; not (only) fixed appliance treatment |
| 244 | Tulloch JFC, Phillips C, Proffit WR. Benefit of early class II treatment: Progress report of a two-phase randomized clinical trial. Am J Orthod Dentofacial Orthop 1998;113(1):62-72. | Excluded; not (only) fixed appliance treatment |
| 245 | Webster T, Harkness M, Herbison P. Associations between changes in selected facial dimensions and the outcome of orthodontic treatment. Am J Orthod Dentofacial Orthop. 1996;110(1):46-53. | Excluded; not (only) fixed appliance treatment |
| 246 | Wijayaratne D, Harkness M, Herbison P. Functional appliance treatment assessed using the PAR index. Aust Orthod J. 2000;16(3):118-26. | Excluded; not (only) fixed appliance treatment |
| 247 | Li W, Wang S, Zhang Y. RETRACTED: The effectiveness of the Invisalign appliance in extraction cases using the the ABO model grading system: a multicenter randomized controlled trial (Retracted Article). International Journal of Clinical and Experimental Medicine. 2015;8(5):8276-82. | Excluded; retracted |
| 248 | Bellini-Pereira SA, Aliaga-Del Castillo A, Vilanova L, de Castro Ribeiro TT, Janson G, Garib D, et al. Treatment stability with bonded versus vacuum-formed retainers after 12 months in adolescents and young adults: A randomized clinical trial*. Orthodontics and Craniofacial Research. 2024. | Excluded; no eligible outcome |
| 249 | Bollen AM, Huang G, King G, Hujoel P, Ma T. Activation time and material stiffness of sequential removable orthodontic appliances. Part 1: Ability to complete treatment. Am J Orthod Dentofacial Orthop. 2003;124(5):496-501. | Excluded; no eligible outcome |
| 250 | Feldmann I. Satisfaction with orthodontic treatment outcome. Angle Orthod. 2014;84(4):581-7. | Excluded; no eligible outcome |
| 251 | Abo Ayach O, Hadad R, Hamadah O. EVALUATION OF EFFICIENCY OF LOW-LEVEL LASER ON RELAPSE AFTER ORTHODONTIC TREATMENT: A RANDOMIZED CONTROLLED CLINICAL TRIAL. Journal of Stomatology. 2021;74(3):140-6. | Included |
| 252 | DiBiase AT, Nasr IH, Scott P, Cobourne MT. Duration of treatment and occlusal outcome using Damon3 self-ligated and conventional orthodontic bracket systems in extraction patients: a prospective randomized clinical trial. Am J Orthod Dentofacial Orthop. 2011;139(2):e111-6. | Included |
| 253 | DiBiase AT, Woodhouse NR, Papageorgiou SN, Johnson N, Slipper C, Grant J, et al. Effects of supplemental vibrational force on space closure, treatment duration, and occlusal outcome: A multicenter randomized clinical trial. Am J Orthod Dentofacial Orthop. 2018;153(4):469-80.e4. | Included |
| 254 | Fleming PS, DiBiase AT, Lee RT. Randomized clinical trial of orthodontic treatment efficiency with self-ligating and conventional fixed orthodontic appliances. Am J Orthod Dentofacial Orthop. 2010;137(6):738-42. | Included |
| 255 | Hegele J, Seitz L, Claussen C, Baumert U, Sabbagh H, Wichelhaus A. Clinical effects with customized brackets and CAD/CAM technology: a prospective controlled study. Progress in Orthodontics. 2021;22(1 C7 - 40). | Included |
| 256 | Jaber ST, Hajeer MY, Burhan AS, Alam MK, Al-Ibrahim HM. Treatment effectiveness of young adults using clear aligners versus buccal fixed appliances in class I malocclusion with first premolar extraction using the ABO-Objective Grading System: A randomized controlled clinical trial. Int Orthod. 2023;21(4):100817. | Included |
| 257 | Jackers N, Maes N, Lambert F, Albert A, Charavet C. Standard vs computer-aided design/computer-aided manufacturing customized self-ligating systems using indirect bonding with both. Angle Orthod. 2021;91(1):74-80. | Included |
| 258 | Jung MH. Effects of self-ligating brackets and other factors influencing orthodontic treatment outcomes: A prospective cohort study. Korean Journal of Orthodontics. 2021;51(6):397-406. | Included |
| 259 | Kaklamanos EG, Mavreas D, Tsalikis L, Karagiannis V, Athanasiou AE. Treatment duration and gingival inflammation in Angle's Class I malocclusion patients treated with the conventional straight-wire method and the Damon technique: a single-centre, randomised clinical trial. J Orthod. 2017;44(2):75-81. | Included |
| 260 | Kumar AG, Bansal A. Effectiveness and acceptability of Essix and Begg retainers: a prospective study. Aust Orthod J. 2011;27(1):52-6. | Included |
| 261 | Lin E, Julien K, Kesterke M, Buschang PH. Differences in finished case quality between Invisalign and traditional fixed appliances. Angle Orthod. 2022;92(2):173-9. | Included |
| 262 | Liu F, Liu L, Wang YH. [Evaluation of the effect of Insignia system in customized orthodontic treatment]. Shanghai Kou Qiang Yi Xue. 2022;31(1):96-9. | Included |
| 263 | Moslemzadeh SH, Sohrabi A, Rafighi A, Farshidnia S. Comparison of Stability of the Results of Orthodontic Treatment and Gingival Health between Hawley and Vacuum-formed Retainers. J Contemp Dent Pract. 2018;19(4):443-9. | Included |
| 264 | Penning EW, Peerlings RHJ, Govers JDM, Rischen RJ, Zinad K, Bronkhorst EM, et al. Orthodontics with Customized versus Noncustomized Appliances: A  Randomized Controlled Clinical Trial. J Dent Res. 2017;96(13):1498-504. | Included |
| 265 | Preston, K.A. (2017) Treatment and post-treatment posterior occlusal changes in Invisalign® and traditional braces: a randomized controlled. MSc thesis, Texas A & M University. | Included |
| 266 | Sandler J, Benson PE, Doyle P, Majumder A, O'Dwyer J, Speight P, et al. Palatal implants are a good alternative to headgear: a randomized trial. Am J Orthod Dentofacial Orthop. 2008;133(1):51-7. | Included |
| 267 | Sandler J, Murray A, Thiruvenkatachari B, Gutierrez R, Speight P, O'Brien K. Effectiveness of 3 methods of anchorage reinforcement for maximum anchorage in  adolescents: A 3-arm multicenter randomized clinical trial. Am J Orthod Dentofacial Orthop. 2014;146(1):10-20. | Included |
| 268 | Sharma V, Sengupta J. Modifications to Increase Efficiency of the Begg Orthodontic Technique. Med J Armed Forces India. 2009 Apr;65(2):118-22. | Included |
| 269 | Yassir YA, El-Angbawi AM, McIntyre GT, Revie GF, Bearn DR. A randomized clinical trial of the effectiveness of 0.018-inch and 0.022-inch slot orthodontic bracket systems: part 1-duration of treatment. Eur J Orthod. 2019 Mar 29;41(2):133-142. | Included |
| 270 | Yassir YA, El-Angbawi AM, McIntyre GT, Revie GF, Bearn DR. A randomized clinical trial of the effectiveness of 0.018-inch and 0.022-inch slot orthodontic bracket  systems: part 2-quality of treatment. Eur J Orthod. 2019;41(2):143-53. | Included |
| 271 | Yıldırım K, Saglam-Aydinatay B. Comparative assessment of treatment efficacy and adverse effects during nonextraction orthodontic treatment of Class I malocclusion patients with direct and indirect bonding: A parallel randomized clinical trial. Am J Orthod Dentofacial Orthop. 2018;154(1):26-34.e1. | Included |

**Appendix 4.** Communication attempts with authors of included studies for additional data.

| **Study** | **Author asked** | **Reason** | **Status** |
| --- | --- | --- | --- |
| Abo Ayach 2021 | Ousama Abo Ayach | Asked for sample and SDs (or dataset) | Response pending |
| Thickett 2010 | None (affiliation change) | - | - |
| Hegele 2021 | Andrea Wichelhaus | Asked for SDs (or dataset) | Response pending |
| Jung 2021 | Min-Ho Jung | Asked for SDs for the ABO-OGS components | Sent SDs |
| Kaklamanos 2017 | Eleftherios Kaklamanos | Asked for SDs of the post-treatment PAR and the data for PAR-reduction and %-PAR-reduction (or dataset) | Sent SDs |
| Sharma 2009 | V Sharma | Asked for SDs (or dataset) | Response pending |
| Jaber 2023 | Samer T Jaber | Asked for SDs of duration (or dataset) | Sent SDs |
| Lin 2022 | Peter Buschang | Asked for means & SD for DI and for dataset (to account for baseline imbalances) | Response pending |
| Preston 2017 | Kathryn Preston | Asked for SDs of the two ABO-OGS components reported in graphs and for any other ABO-OGS components (or dataset) | Response pending |

ABO, American Board of Orthodontics; OGS, objective grading system; PAR, peer assessment rating; SD, standard deviation.

**Appendix 5.** Risk of bias of included randomized trials with the RoB 2 tool.

| **Nr** | **Study** | **Randomization process** | **Deviations from intended interventions** | **Missing outcome data** | **Measurement of the outcome** | **Selection of the reported result** | **Overall** |
| --- | --- | --- | --- | --- | --- | --- | --- |
| 1 | Abo Ayach 2021 | **High risk**  **(non-randomized for Tx)** | **Low risk** | **Low risk** | **High risk**  **(partial blinding; no calibration)** | **Some concerns** | **High risk** |
| 2 | DiBiase 2011 | **Low risk** | **Low risk** | **Low risk** | **High risk**  **(no blinding; no calibration)** | **Some concerns** | **High risk** |
| 3 | DiBiase 2018 | **Low risk** | **Low risk** | **Low risk (similar attrition)** | **Low risk**  **(blinding; calibration)** | **Some concerns** | **Low risk** |
| 4 | Fleming 2010 | **Low risk** | **Low risk** | **Low risk (similar attrition)** | **High risk**  **(no blinding; no calibration)** | **Some concerns** | **High risk** |
| 5 | Hegele 2021 | **High risk**  **(quasi-randomization)** | **Low risk** | **Low risk**  **(small attrition)** | **High risk**  **(no blinding; no calibration)** | **Some concerns** | **High risk** |
| 6 | Jaber 2023 | **Low risk** | **No information** | **Low risk (similar attrition)** | **Low risk**  **(blinding; calibration)** | **Some concerns** | **Low risk** |
| 7 | Jackers 2021 | **High risk**  **(previous randomization)** | **Low risk** | **Low risk** | **High risk**  **(no blinding; no calibration)** | **Some concerns** | **High risk** |
| 8 | Jung 2021 | **High risk**  **(quasi-randomization)** | **Low risk** | **Low risk (similar attrition)** | **Some concerns (blinding; no calibration)** | **Some concerns** | **High risk** |
| 9 | Kaklamanos 2917 | **Low risk** | **Low risk** | **Low risk** | **Low risk**  **(blinding; calibration)** | **Some concerns** | **Low risk** |
| 10 | Kumar 2011 | **High risk**  **(non-randomized for Tx)** | **Low risk** | **Low risk** | **High risk**  **(no blinding; no calibration)** | **Some concerns** | **High risk** |
| 11 | Lin 2022 | **High risk**  **(baseline imbalance)** | **No information** | **High risk**  **(unequal / high attrition)** | **Low risk**  **(blinding; calibration)** | **Some concerns** | **High risk** |
| 12 | Liu 2022 | **High risk**  **(baseline imbalance)** | **Low risk** | **Low risk** | **High risk**  **(no blinding; no calibration)** | **Some concerns** | **High risk** |
| 13 | Moslemzadeh 2018 | **High risk**  **(non-randomized for Tx)** | **Low risk** | **Low risk** | **Some concerns (blinding; no calibration)** | **Some concerns** | **High risk** |
| 14 | Penning 2017 | **Low risk** | **Low risk** | **Low risk (similar attrition)** | **Low risk**  **(blinding; calibration)** | **Some concerns** | **Low risk** |
| 15 | Preston 2017 | **Low risk** | **No information** | **High risk**  **(unequal / high attrition)** | **High risk**  **(no blinding; no calibration)** | **Some concerns** | **High risk** |
| 16 | Sandler 2008 | **Low risk** | **Low risk** | **Low risk (similar attrition)** | **High risk**  **(no blinding; no calibration)** | **Some concerns** | **High risk** |
| 17 | Sandler 2014 | **Low risk** | **Low risk** | **Low risk** | **Low risk**  **(blinding; calibration)** | **Some concerns** | **Low risk** |
| 18 | Sharma 2010 | **No information** | **Low risk** | **Low risk** | **High risk**  **(no blinding; no calibration)** | **Some concerns** | **High risk** |
| 19 | Yassir 2019 | **Low risk** | **Low risk** | **Low risk**  **(high / equal attrition)** | **Low risk**  **(blinding; calibration)** | **Some concerns** | **Low risk** |
| 20 | Yildirim 2018 | **Low risk** | **Low risk** | **Low risk** | **Low risk**  **(blinding; calibration)** | **Some concerns** | **Low risk** |

Tx, treatment.

**Appendix 6.** Trial arms from each trial included in the single-group (pooled as one arm; marked with green font) or trial arms compared in pairwise (2-group) meta-analyses (blue and red font).

| **Study** | **Scope** | **Single-group meta-analysis** | **Pairwise (2-group) meta-analysis** |
| --- | --- | --- | --- |
| Abo Ayach  2021 | Retention | **G1: BLR G2: VFR**  **G3: VFR + LLLT** | G1: BLR G2: VFR  G3: VFR + LLLT |
| DiBiase 2011 | FXA | **G1: Labial FXA G2: Labial SL FXA** | **G1: Labial FXA G2: Labial SL FXA** |
| DiBiase 2018 | FXA | **G1: Labial FXA**  G2: Labial FXA + VIB G3: Labial FXA + PLB | **G1: Labial FXA**  G2: Labial FXA + VIB  **G3: Labial FXA + PLB** |
| Fleming 2010 | FXA | **G1: Labial FXA G2: Labial SL FXA** | **G1: Labial FXA G2: Labial SL FXA** |
| Hegele 2021 | FXA | **G1: Labial SL FXA**  **G2: Labial CAD SL FXA** | **G1: Labial SL FXA**  **G2: Labial CAD SL FXA** |
| Jaber 2023 | FXA / aligners | **G1: Labial FXA**  G2: Aligners | **G1: Labial FXA G2: Aligners** |
| Jackers  2021 | FXA | **G1: Labial SL FXA**  **G2: Labial CAD SL FXA** | **G1: Labial SL FXA**  **G2: Labial CAD SL FXA** |
| Jung 2021 | FXA | **G1: Labial Cer FXA G2: Labial SL Cer FXA** | **G1: Labial Cer FXA G2: Labial SL Cer FXA** |
| Kaklama nos 2917 | FXA | **G1: Labial FXA G2: Labial SL FXA** | **G1: Labial FXA G2: Labial SL FXA** |
| Kumar  2011 | Retention | **G1: Essig retainer**  **G2: Begg retainer** | G1: Essig retainer  G2: Begg retainer |
| Lin 2022 | FXA / aligners | **G1: Labial FXA**  G2: Aligners | **G1: Labial FXA G2: Aligners** |
| Liu 2022 | FXA | **G1: Labial SL FXA**  **G2: Labial CAD SL FXA** | **G1: Labial SL FXA**  **G2: Labial CAD SL FXA** |
| Moslemz  adeh 2018 | Retention | **G1: VFR (1.0 mm)**  **G2: VFR (1.5 mm)**  **G3: Hawley retainer** | G1: VFR (1.0 mm)  G2: VFR (1.5 mm)  G3: Hawley retainer |
| Penning 2017 | FXA | **G1: Labial SL FXA**  **G2: Labial CAD SL FXA** | **G1: Labial SL FXA**  **G2: Labial CAD SL FXA** |
| Preston  2017 | FXA / aligners | **G1: Labial FXA**  G2: Aligners | **G1: Labial FXA**  **G2: Aligners** |
| Sandler 2008 | FXA | **G1: Labial FXA + HG G2: Labial FXA + TAD** | **G1: Labial FXA + HG G2: Labial FXA + TAD** |
| Sandler 2014 | FXA | **G1: Labial FXA + HG G2: Labial FXA + Nance**  **G3: Labial FXA + TAD** | **G1: Labial FXA + HG G2: Labial FXA + Nance**  **G3: Labial FXA + TAD** |
| Sharma 2010 | FXA | **G1: Labial FXA (Begg)**  **G2: Labial FXA (modified Begg) G3: Labial FXA (straightwire)** | G1: Labial FXA (Begg)  G2: Labial FXA (modified Begg) G3: Labial FXA (straightwire) |
| Yassir  2019coll | FXA | **G1: Labial FXA (0.022” slot) G2: Labial FXA (0.018” slot)** | **G1: Labial FXA (0.022” slot) G2: Labial FXA (0.018” slot)** |
| Yildirim 2018 | FXA | **G1: Labial FXA (directly bonded) G2: Labial FXA (indirectly bonded)** | **G1: Labial FXA (directly bonded) G2: Labial FXA (indirectly bonded)** |

BLR, bonded lingual/palatal retainer; CAD, computer-assisted designed / manufactured; Cer, ceramic; coll, multiple trial reports collated; FXA, conventional fixed appliances (brackets); LLLT, low-level laser therapy; PLB, placebo; SL, self-ligating; TAD, temporary anchorage device; VFR, vacuum formed retainer; VIB, vibration supplement.

**Appendix 7.** Outcomes assessed by only one trial each.

| **Nr** | **Comparison** | **Outcome** | **MD (95% CI)** | **P** | **Clinically relevant*** | **Interpretation** |
| --- | --- | --- | --- | --- | --- | --- |
| 1 | 0.018” vs 0.022” slot brackets | PAR post-Tx | 1.40 (-0.16, 2.96) | 0.07 | - |  |
| 2 |  | % PAR reduction | -3.00 (-8.95, 2.95) | 0.32 | - |  |
| 3 |  | ABO-OGS total post-Tx | 0.20 (-3.15, 3.55) | 0.91 | - |  |
| 4 |  | ABO-OGS: alignment/rotations | -0.50 (-1.48, 0.48) | 0.32 | - |  |
| 5 |  | ABO-OGS: marginal ridges | -0.80 (-1.60, 0.00) | 0.05 | - |  |
| 6 |  | ABO-OGS: buccolingual inclination | 0.00 (-0.99, 0.99) | 1.00 | - |  |
| 7 |  | ABO-OGS: overjet | 0.50 (-0.54, 1.54) | 0.35 | - |  |
| 8 |  | ABO-OGS: occlusal contacts | 0.20 (-1.20, 1.60) | 0.78 | - |  |
| 9 |  | ABO-OGS: occlusal relationships | 0.50 (-0.62, 1.62) | 0.38 | - |  |
| 10 |  | ABO-OGS: interproximal contacts | 0.30 (-0.13, 0.73) | 0.17 | - |  |
| 11 |  | Tx duration | -1.90 (-5.39, 1.59) | 0.29 | - |  |
|  |  |  |  |  |  |  |
| 12 | Aligners vs fixed appliances | Tx duration | 2.40 (-0.93, 5.73) | 0.16 | - |  |
|  |  |  |  |  |  |  |
| 13 | CAD/CAM vs pre-fabricated brackets | PAR reduction | 1.76 (-0.66, 4.18) | 0.15 | - |  |
| 14 |  | % PAR reduction | 4.00 (-0.76, 8.76) | 0.09 | - |  |
| 15 |  | ABO-OGS total post-Tx | -4.10 (-9.06, 0.86) | 0.11 | - |  |
| 16 |  | ABO-OGS: alignment/rotations | 0.00 (-0.92, 0.92) | 1.00 | - |  |
| 17 |  | ABO-OGS: marginal ridges | 0.20 (-1.36, 1.76) | 0.80 | - |  |
| 18 |  | ABO-OGS: buccolingual inclination | 0.00 (-1.76, 1.76) | 1.00 | - |  |
| 19 |  | ABO-OGS: overjet | -1.30 (-2.98, 0.38) | 0.13 | - |  |
| 20 |  | ABO-OGS: occlusal contacts | -3.30 (-6.03, -0.57) | 0.01 | Yes | Improved occlusal contacts with CAD/CAM brackets |
| 21 |  | ABO-OGS: occlusal relationships | -0.50 (-2.91, 1.91) | 0.68 | - |  |
| 22 |  | ABO-OGS: interproximal contacts | 0.50 (-0.04, 1.04) | 0.06 | - |  |
| 23 |  | ABO-OGS: root angulation | 0.35 (-0.31, 1.01) | 0.30 | - |  |
|  |  |  |  |  |  |  |
| 24 | Indirectly vs directly bonded brackets | ABO-OGS total post-Tx | -5.00 (-8.04, -1.96) | 0.001 | Yes | Improved finishing quality with indirectly bonded brackets |
| 25 |  | ABO-OGS: alignment/rotations | 0.00 (-1.89, 1.89) | 1.00 | - |  |
| 26 |  | ABO-OGS: marginal ridges | -2.00 (-3.89, -0.11) | 0.03 | Yes | Improved marginal ridges with indirectly bonded brackets |
| 27 |  | ABO-OGS: buccolingual inclination | 0.00 (-1.36, 1.36) | 1.00 | - |  |
| 28 |  | ABO-OGS: overjet | -1.00 (-2.07, 0.07) | 0.06 | - |  |
| 29 |  | ABO-OGS: occlusal contacts | 0.00 (-1.20, 1.20) | 1.00 | - |  |
| 30 |  | ABO-OGS: occlusal relationships | 0.00 (-0.39, 0.39) | 1.00 | - |  |
| 31 |  | ABO-OGS: interproximal contacts | 0.00 (-0.14, 0.14) | 1.00 | - |  |
| 32 |  | ABO-OGS: root angulation | 0.00 (-1.36, 1.36) | 1.00 | - |  |
| 33 |  | Tx duration | -0.60 (-2.58, 1.38) | 0.55 | - |  |
|  |  |  |  |  |  |  |
| 34 | SL vs conventionally-ligated brackets | ABO-OGS total post-Tx | 2.74 (1.14, 4.34) | 0.001 | Yes | Worse finishing quality with self-ligating brackets |
| 35 |  | ABO-OGS: alignment/rotations | 0.34 (-0.04, 0.72) | 0.08 | - |  |
| 36 |  | ABO-OGS: marginal ridges | -0.02 (-0.36, 0.32) | 0.91 | - |  |
| 37 |  | ABO-OGS: buccolingual inclination | 1.51 (0.84, 2.18) | <0.001 | Yes | Worse buccolingual inclination with self-ligating brackets |

| 38 |  | ABO-OGS: overjet | -0.24 (-0.63, 0.15) | 0.23 | - |  |
| --- | --- | --- | --- | --- | --- | --- |
| 39 |  | ABO-OGS: occlusal contacts | 0.38 (-0.48, 1.24) | 0.39 | - |  |
| 40 |  | ABO-OGS: occlusal relationships | 0.44 (-0.46, 1.34) | 0.34 | - |  |
| 41 |  | ABO-OGS: interproximal contacts | 0.03 (-0.05, 0.11) | 0.49 | - |  |
| 42 |  | ABO-OGS: root angulation | 0.30 (0.04, 0.57) | 0.02 | No | No clinically relevant difference in root angulation |
| 43 |  | PAR reduction | -3.54 (-9.35, 2.27) | 0.23 | - |  |
|  |  |  |  |  |  |  |
| 44 | Vibration adjunct vs control | PAR post-Tx | 0.00 (-1.39, 1.39) | 1.00 | - |  |
| 45 |  | PAR reduction | -0.35 (-1.60, 0.90) | 0.58 | - |  |
| 46 |  | % PAR reduction | -1.54 (-5.11, 2.03) | 0.40 | - |  |
| 47 |  | Tx duration | 2.19 (-0.69, 5.07) | 0.14 | - |  |

ABO, American Board of Orthodontics; CAD/CAM, computer-assisted designed / manufactured; CI, confidence interval; MD, mean difference; OGS, objective grading system; PAR, peer assessment rating; SL, self-ligating; Tx, treatment.

* defined as difference larger than half a standard deviation of the control group.

**Appendix 8.** Meta-regressions of single-group meta-analyses of averages.

| **Outcome** | **Covariate** | **Studies** | **Coefficient (95% CI)** | **PMR** |
| --- | --- | --- | --- | --- |
| PAR post-Tx | Pre-Tx age (per year) | 9 | 0.14 (-0.95, 1.22) | 0.77 |
|  | Pre-Tx % male (per 10%) | 8 | 0.03 (-1.56, 1.62) | 0.96 |
|  | Pre-Tx PAR (per point) | 9 | 0.21 (-0.11, 0.53) | 0.17 |
|  | Tx duration (per month) | 9 | 0.32 (-0.03, 0.68) | 0.06 |
|  |  |  |  |  |
| PAR reduction | Pre-Tx age (per year) | - | Not calculable |  |
|  | Pre-Tx % male (per 10%) | 4 | Not calculable |  |
|  | Pre-Tx PAR (per point) | 5 | 0.64 (0.04, 1.23) | 0.04 |
|  | Tx duration (per month) | 5 | 0.55 (-0.75, 1.85) | 0.27 |
|  |  |  |  |  |
| % PAR reduction | Pre-Tx age (per year) | 6 | -2.17 (-9.51, 5.16) | 0.46 |
|  | Pre-Tx % male (per 10%) | 5 | 6.90 (-0.54, 14.34) | 0.05 |
|  | Pre-Tx PAR (per point) | 6 | -0.35 (-2.25, 1.56) | 0.64 |
|  | Tx duration (per month) | 6 | -0.78 (-3.05, 1.49) | 0.39 |
|  |  |  |  |  |
| ABO-OGS total post-Tx | Pre-Tx age (per year) | 7 | -0.14 (-2.90, 2.62) | 0.90 |
|  | Pre-Tx % male (per 10%) | 5 | -1.08 (-27.70, 25.54) | 0.91 |
|  | Pre-Tx PAR (per point) | 1 | Not calculable |  |
|  | Tx duration (per month) | 6 | 0.37 (-0.71, 1.44) | 0.40 |
|  |  |  |  |  |
| Tx duration | Pre-Tx age (per year) | 14 | 0.62 (-0.51, 1.75) | 0.26 |
|  | Pre-Tx % male (per 10%) | 12 | 0.05 (-3.22, 3.32) | 0.97 |
|  | Pre-Tx PAR (per point) | 9 | 0.61 (0.14, 1.09) | 0.01 |

ABO, American Board of Orthodontics; CI, confidence interval; OGS, objective grading system; PAR, peer assessment rating; PMR, p value from meta-regression (significant if <0.10); Tx, treatment.

**Appendix 9.** Contour-enhanced funnel plot for the single-group meta-analysis of average post-treatment PAR.

Standard Error

0.706

0.471

**Post-Tx PAR (Thompson test P=0.02)**

0.236

0


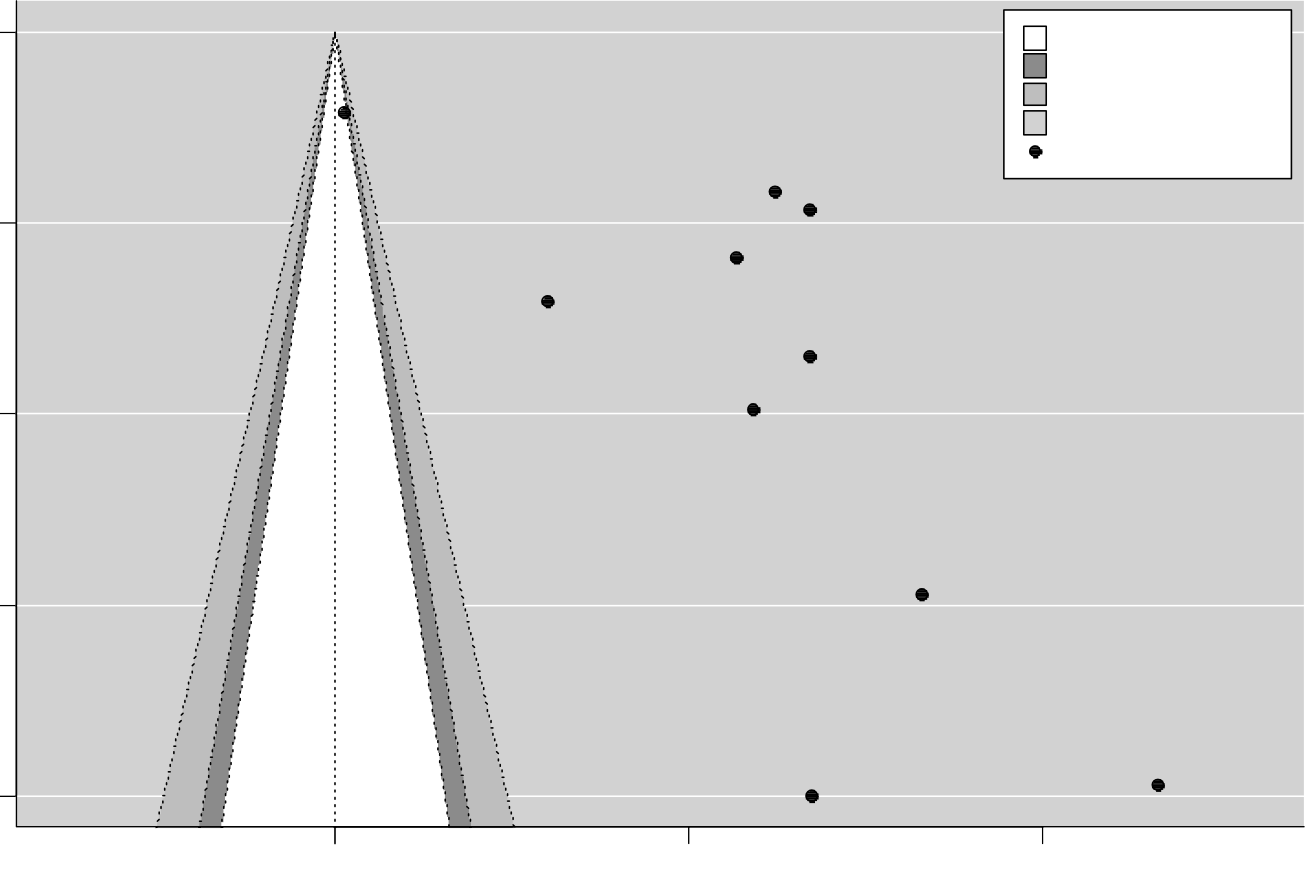


0.10 < p 1.00

0.05 < p 0.10

0.01 < p 0.05

0.00 < p 0.01

Studies

0

5

10

# Observed Outcome

0.942

**Appendix 10.** Contour-enhanced funnel plot for the single-group meta-analysis of average treatment duration.

Standard Error

2.076

**Treatment duration (Thompson test P=0.31)**

1.038

0


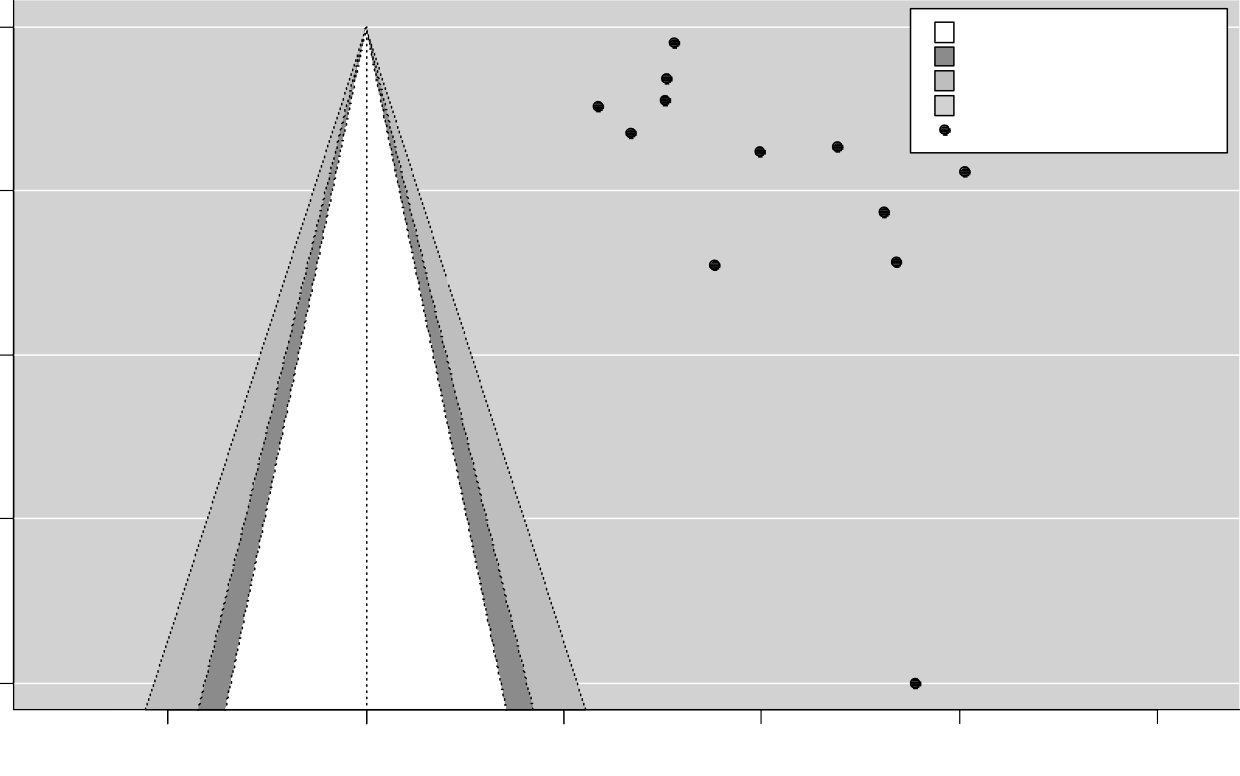


0.10 < p 1.00

0.05 < p 0.10

0.01 < p 0.05

0.00 < p 0.01

S t u d i e s

-10

0

10

20

30

40

Observed Outcome

4.152

3.114

**Appendix 11.** Sensitivity analysis of single-group meta-analysis of average post-treatment PAR by precision.

|  |  | **50% most precise** |  | **50% least precise** | **Pbetween-subset** |
| --- | --- | --- | --- | --- | --- |
| **Outcome** | **n** | **Mean (95% CI)** | **n** | **Mean (95% CI)** |  |
| PAR post-treatment | 5 | 4.34 (0.92, 7.77) | 5 | 7.78 (5.00, 10.56) | 0.03 |

ABO, American Board of Orthodontics; OGS, objective grading system; PAR, peer assessment rating; Tx, treatment.

**Appendix 12 .** Sensitivity analysis of pairwise (2-group) meta-analyses by including only trials with low risk of bias.

|  |  | **All trials** | | |  | **Low risk trials** | | |
| --- | --- | --- | --- | --- | --- | --- | --- | --- |
| **Comparison** | **Outcome** | **n** | **MD (95% CI)** | **P** |  | **n** | **MD (95% CI)** | **P** |
| Aligners vs fixed appliances | ABO-OGS total post-Tx | 2 | 0.05 (-9.36, 9.46) | 0.99 |  | 1 | 4.61 (0.18, 9.04) | 0.04 |
|  | Tx duration | 2 | -0.22 (-5.44, 5.00) | 0.93 |  | 1 | -0.63 (-4.22, 0.66) | 0.73 |
|  |  |  |  |  |  |  |  |  |
| CAD/CAM vs pre-fabricated brackets | PAR post-Tx | 2 | -0.15 (-0.78, 0.48) | 0.64 |  | 1 | -0.55 (-1.64, 0.54) | 0.32 |
|  | Tx duration | 2 | -1.43 (-5.42, 2.55) | 0.48 |  | 1 | 0.60 (-0.68, 1.88) | 036 |
|  |  |  |  |  |  |  |  |  |
| Skeletal vs conventional anchorage | PAR post-Tx | 2 | -1.36 (-5.29, 2.57) | 0.50 |  | 1 | -3.36 (-5.87, -0.85) | 0.008 |
|  | PAR reduction | 2 | 1.09 (-3.56, 5.73) | 0.65 |  | 1 | 2.98 (-3.59, 9.55) | 0.37 |
|  | Tx duration | 2 | -0.96 (-4.98, 3.07) | 0.64 |  | 1 | -0.87 (-20.93, 19.18) | 0.93 |
|  |  |  |  |  |  |  |  |  |
| SL vs conventionally-ligated brackets | PAR post-Tx | 3 | 0.23 (-0.92, 1.38) | 0.70 |  | 1 | 0.09 (-0.31, 0.49) | 0.66 |
|  | % PAR reduction | 3 | -0.42 (-2.04, 1.20) | 0.62 |  | 1 | -0.52 (-2.24, 1.20) | 0.55 |
|  | Tx duration | 4 | -0.08 (-2.36, 2.21) | 0.95 |  | 1 | -2.25 (-4.74, 0.24) | 0.07 |

ABO, American Board of Orthodontics; CAD/CAM, computer-assisted designed / manufactured; CI, confidence interval; MD, mean difference; OGS, objective grading system; PAR, peer assessment rating; SL, self-ligating; Tx, treatment.
